# Halogen-Bond Mediated [2+2] Photodimerizations: À la Carte Access to Unsymmetrical Cyclobutanes in the Solid State

**DOI:** 10.3390/molecules27031048

**Published:** 2022-02-03

**Authors:** Jay Quentin, Eric W. Reinheimer, Leonard R. MacGillivray

**Affiliations:** 1 Department of Chemistry, University of Iowa, Iowa City, IA 52242, USA; jay-bell@uiowa.edu; 2 Rigaku Americas Corporation, 9009 New Trails Drive, The Woodlands, TX 77381, USA; eric.reinheimer@rigaku.com

**Keywords:** cocrystal, crystal engineering, halogen bonding, photodimerization, cyclobutane

## Abstract

The ditopic halogen-bond (X-bond) donors 1,2-, 1,3-, and 1,4-diiodotetrafluorobenzene (**1,2-**, **1,3-**, and **1,4-di-I-tFb**, respectively) form binary cocrystals with the unsymmetrical ditopic X-bond acceptor *trans*-1-(2-pyridyl)-2-(4-pyridyl)ethylene (**2,4-bpe**). The components of each cocrystal (**1,2-di-I-tFb**)·(**2,4-bpe**), (**1,3-di-I-tFb**)·(**2,4-bpe**), and (**1,4-di-I-tFb**)·(**2,4-bpe**) assemble via N···I X-bonds. For (**1,2-di-I-tFb**)·(**2,4-bpe**) and (**1,3-di-I-tFb**)·(**2,4-bpe**), the X-bond donor supports the C=C bonds of **2,4-bpe** to undergo a topochemical [2+2] photodimerization in the solid state: UV-irradiation of each solid resulted in stereospecific, regiospecific, and quantitative photodimerization of **2,4-bpe** to the corresponding head-to-tail (*ht*) or head-to-head (*hh*) cyclobutane photoproduct, respectively.

## 1. Introduction

Cyclobutane rings appended with *n*-pyridyl (*n* = 2, 3 or 4) (pyr) groups are useful building blocks to construct metal-organic assemblies and materials [1,2,3,4]. Many such molecules have been accessed via template-directed, topochemical [2+2] photodimerizations of alkenes within cocrystals. These transformations are conducted in the organic solid state and consequently, due to the highly ordered environment characteristic of crystalline reaction media, often proceed stereospecifically and quantitatively. Of particular and recent interest to our group have been cyclobutanes derived from photodimerization of unsymmetrical alkenes. These photoproducts are appended with two pairs of differently substituted pyr groups. Head-to-head (*hh*) and head-to-tail (*ht*) regioisomers are possible from photodimerizations of unsymmetrical alkenes [5]. Given that covalent-bond-forming reactions performed in the solid state are extremely sensitive to molecular packing, it is imperative to identify diverse and robust classes of template molecules capable of directing photodimerizations in crystals.

**Scheme 1:**
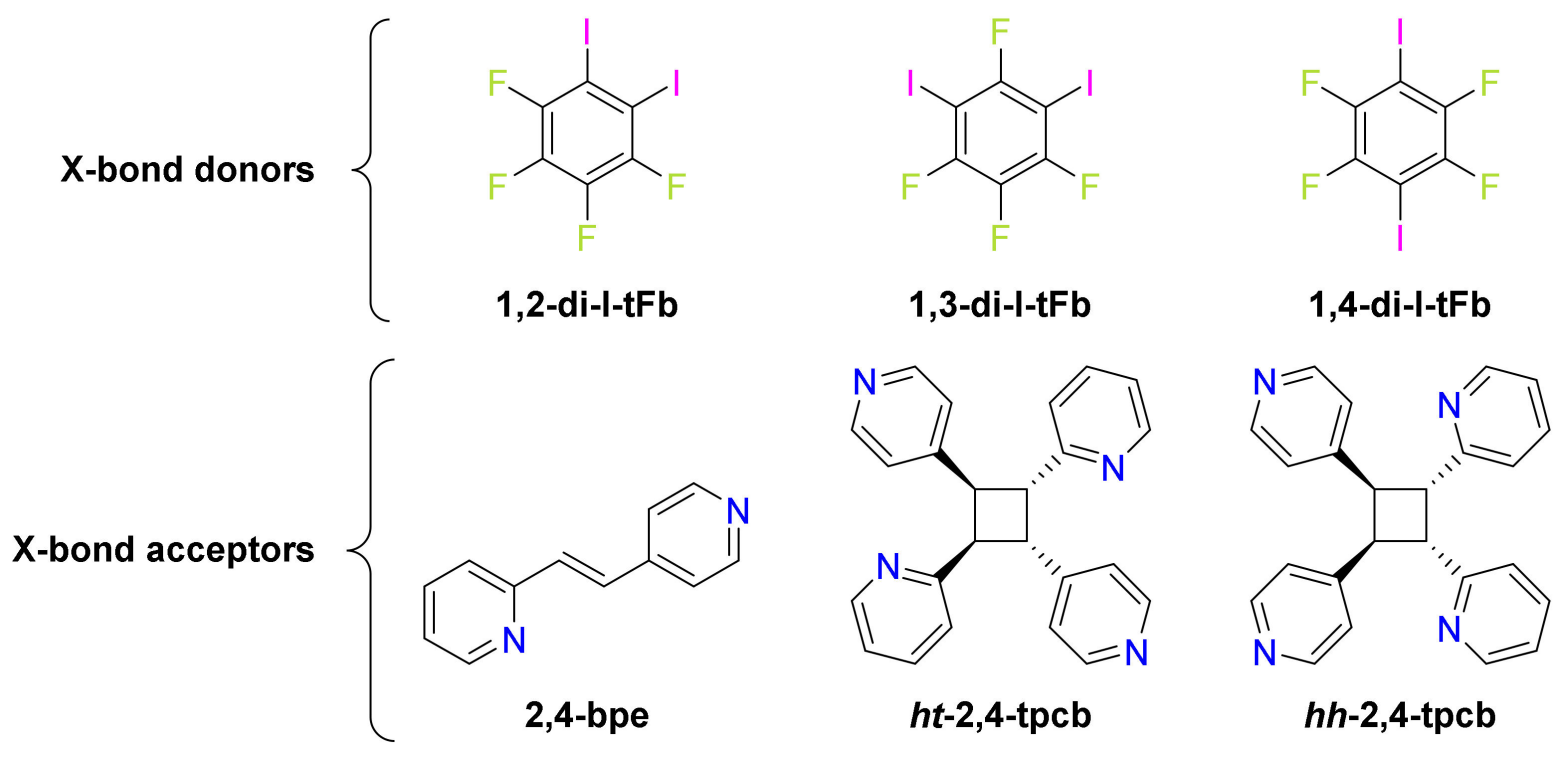
Ditopic components for cocrystals and photoproducts.

**Scheme 2:**
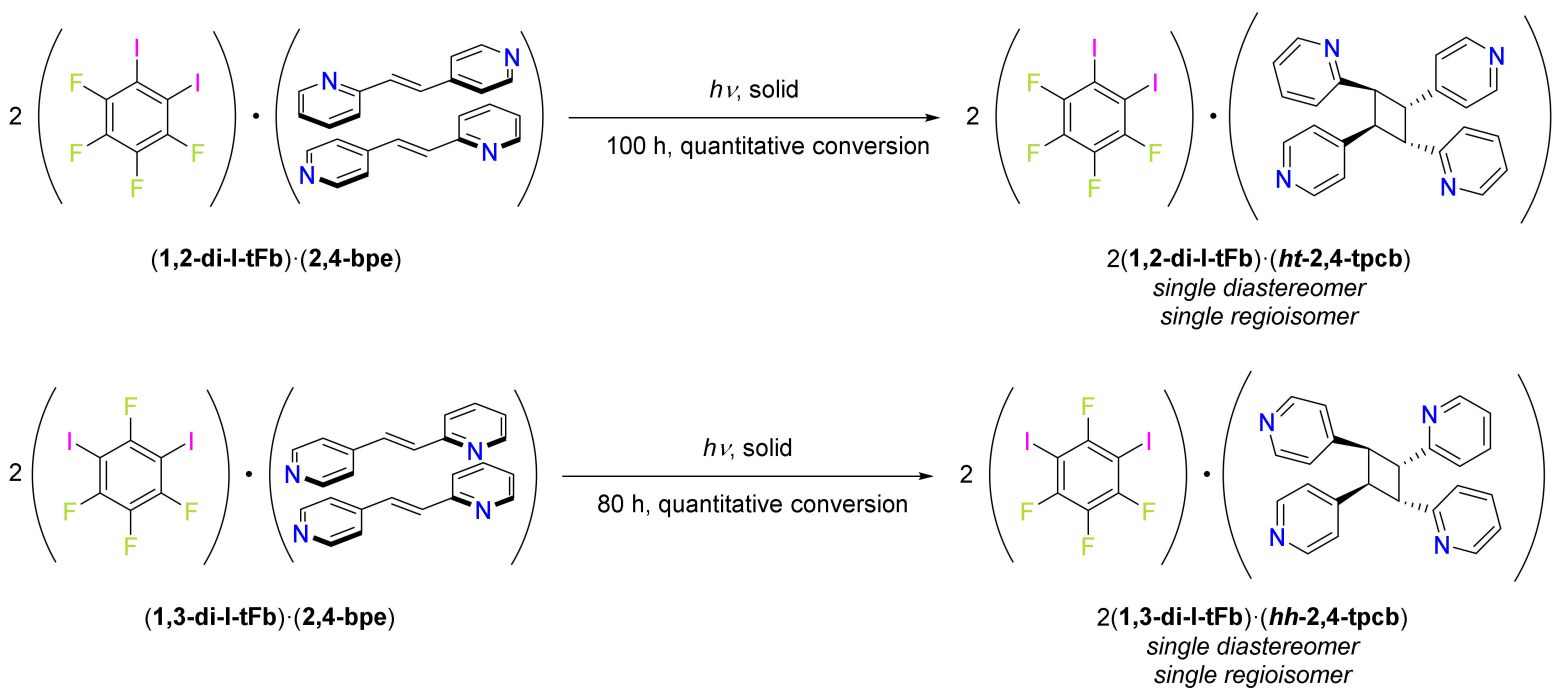
UV-irradiation of (**1,2-di-I-tFb**)·(**2,4-bpe**) or (**1,3-di-I-tFb**)·(**2,4-bpe**) generates either ***ht*-** or ***hh*-2,4-tpcb**, respectively.

Herein, we report a series of binary cocrystals comprising components that self-assemble via N∙∙∙I X-bonds. We show that in two of these cocrystals, the ditopic X-bond donor serves to support nearest-neighbor unsymmetrical alkenes (the X-bond acceptors) in a geometry conducive to topochemical [2+2] photodimerization in the solid state. Evidence is rapidly emerging that demonstrates X-bonds as useful supramolecular synthons in cocrystals to support [2+2] photodimerizations of alkenes appended with pyr groups. Whereas there are several examples of photodimerizations of symmetrical alkenes mediated by X-bonds [6,7,8,9,10,11], we are aware of only one example – as reported by Groeneman [12] – wherein an unsymmetrical alkene is assembled to react via X-bonds. We report on the single-crystal X-ray structures of the binary cocrystals (**1,*n*-di-I-tFb**)·(**2,4-bpe**) (*n* = 2, 3 or 4), 2(**1,2-di-I-tFb**)·(***ht*-2,4-tpcb**), and 2(**1,3-di-I-tFb**)·(***hh*-2,4-tpcb**) (Scheme 1) with components sustained by N∙∙∙I X-bonds. For (**1,2-di-I-tFb**)·(**2,4-bpe**) and (**1,3-di-I-tFb**)·(**2,4-bpe**), we show the unsymmetrical alkene to generate the regioisomers *rctt*-1,3-bis(2-pyridyl)-2,4-bis(4-pyridyl)cyclobutane (***ht*-2,4-tpcb**) and *rctt*-1,2-bis(2-pyridyl)-3,4-bis(4-pyridyl)cyclobutane (***hh*-2,4-tpcb**), respectively, stereospecifically, regiospecifically, and in quantitative conversion (Scheme 2).

## 2. Results and Discussion

Work by our group has demonstrated that the unsymmetrical cyclobutanes *rctt*-bis(*n*-pyridyl)-bis(*n′*-pyridyl)cyclobutanes (*n* ≠ *n′*, *n* = 2 or 4, *n′* = 2 or 4) can be constructed in the solid state by way of hydrogen-bond (H-bond) mediated self-assembly. The photoproducts were generated using ditopic H-bond donor coformers in binary cocrystals. Cyclobutanes with the pyr substituents in both *ht*- [13] and *hh*-regiochemistries [14] were obtained via infinite and discrete H-bonded assemblies, respectively. While H- and X- bonds often display similar structural effects in the solid state (i.e., strength, directionality), the donor moieties (e.g., hydroxyl versus halogen) exhibit very different chemical properties, which can impact processes that follow the solid-state reactions (e.g., separations of photoproducts) [15].

### 2.1. X-ray Crystal Structure of *(**1,2-di-I-tFb**)∙(**2,4-bpe**)*

**Figure 1:**
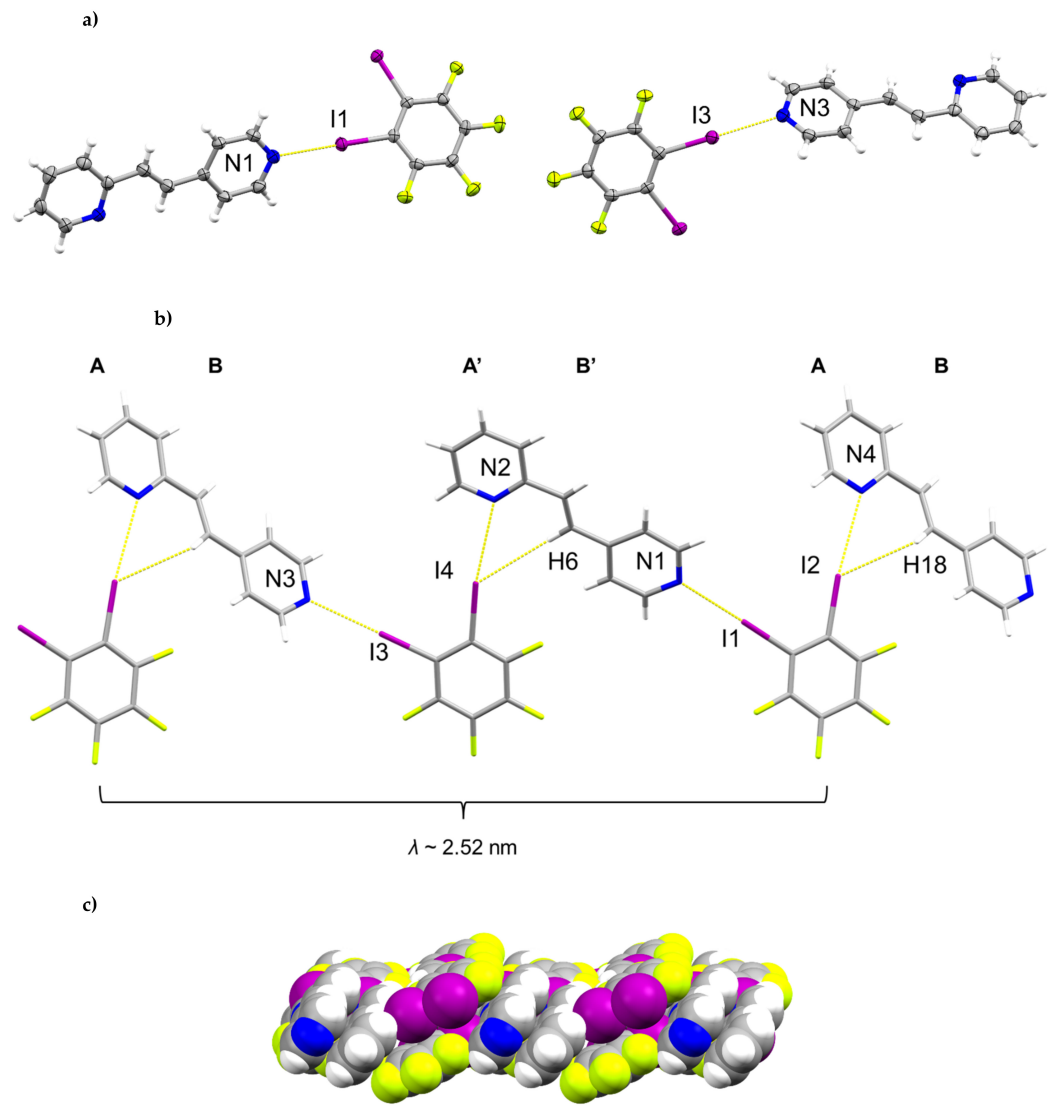

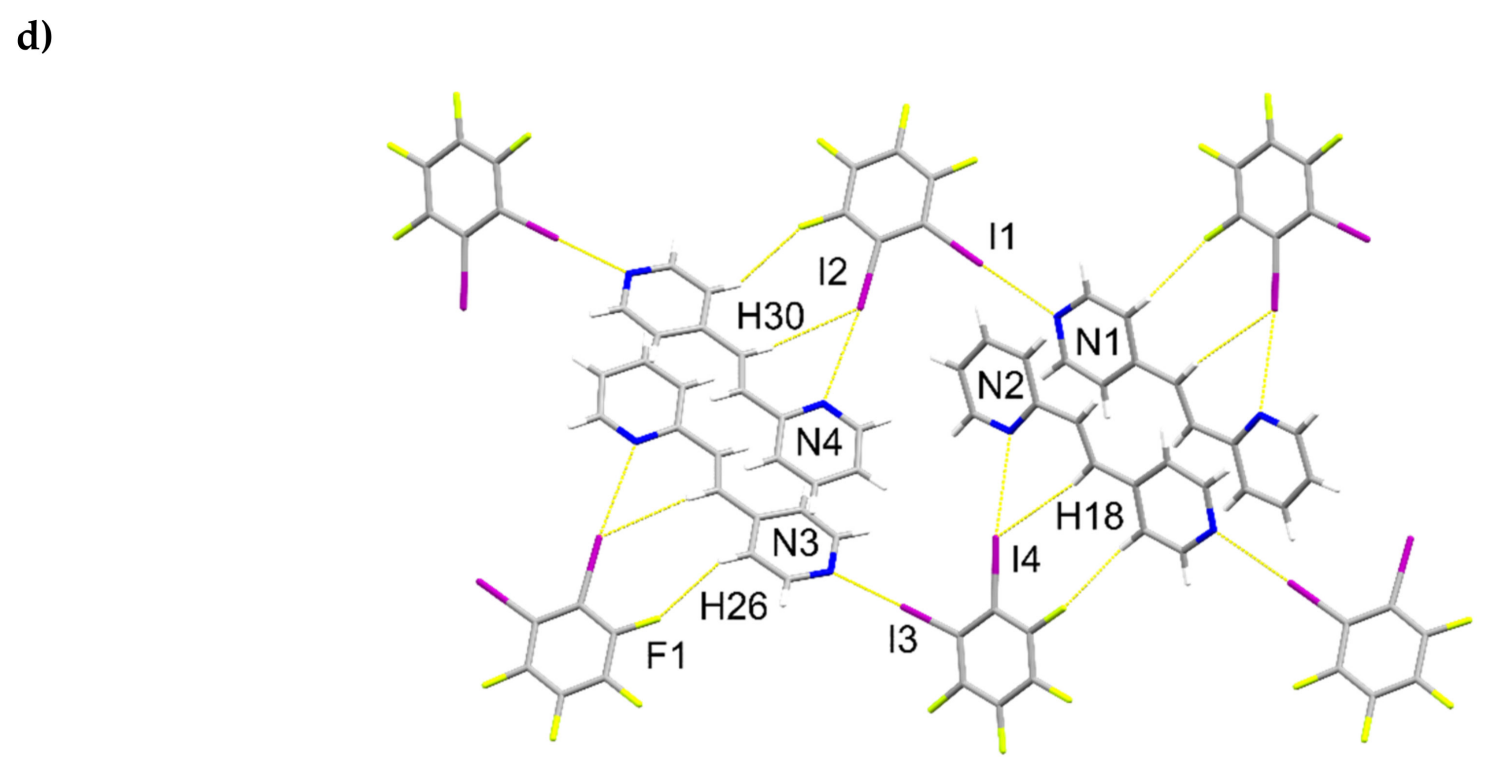
Perspectives of (**1,2-di-I-tFb**)∙(**2,4-bpe**): (**a**) asymmetric unit (anisotropic displacement ellipsoids at 50% probability; view along *a*); (**b**) 1D tapes illustrating ABA’B’ repeat motif (view along *a*); (**c**) corrugated layered structure (space-filling); and (**d**) reactive arrangement of nearest-neighbor alkene pairs.

The components of (**1,2-di-I-tFb**)∙(**2,4-bpe**) crystallize in the triclinic space group *P*$\bar{1}$ (Figure 1, Table 1). The asymmetric unit consists of two unique molecules each of **1,2-di-I-tFb** and **2,4-bpe** (Figure 1a). The pyr rings of the two molecules of **2,4-bpe** lie approximately coplanar and exhibit different twist angles (*ϕ* ~ 3.4° for pyr_N1/N2_, *ϕ* ~ 8.0° for pyr_N3/N4_, Table 2). The components of the cocrystal interact primarily via N∙∙∙I X-bonds (*d*(N1∙∙∙I1) = 2.808(2) Å; *d*(N2∙∙∙I4) = 3.147(2) Å; *d*(N3∙∙∙I3) = 2.814(2) Å); *d*(N4∙∙∙I2) = 3.054(2) Å, Table 3), as well as secondary C-H∙∙∙F forces (*d*(H18∙∙∙I2) ~ 2.98 Å; *d*(H6∙∙∙I4) ~ 2.98 Å). Because of the arrangement, the components form 1D tapes (*λ* ~ 2.52 nm) along a diagonal within the crystallographic *bc*-plane (Figure 1b, Table 4). Adjacent chains run parallel and exhibit a tongue-in-groove fit manifested approximately along the crystallographic *b*-axis to give a corrugated, 2D, layered structure (Figure 1c, Table 4). Chains within adjacent layers run antiparallel. Alkenes between layers stack face-to-face and antiparallel (*ht*) with nearest-neighbor C=C bonds of **2,4’-bpe** separated by 3.80 Å [N1/N2] and 3.72 Å [N3/N4] (Figure 1d). Both arrangements conform to the criteria of Schmidt [16] for topochemical [2+2] photodimerization.

The alkene C=C bonds of (**1,2-di-I-tFb**)∙(**2,4-bpe**) are photoactive. UV-irradiation (broadband Hg lamp, 100 h) of the cocrystal (**1,2-di-I-tFb**)·(**2,4-bpe**) resulted in stereospecific, regiospecific, and quantitative photodimerization of **2,4-bpe** to generate ***ht*-2,4-tpcb** (Scheme 2, Table 4) [17]. The formation of the cyclobutane photoproduct was evidenced by the complete disappearance of the pair of olefinic doublets (*δ*_H_ = 7.65, 7.59 ppm) with concomitant emergence of a pair of cyclobutane resonances (*δ*_H_ = 4.87 − 4.82, 4.79 − 4.74 ppm) in the ^1^H NMR spectrum (Figure S1 of the ESI).

### 2.2. X-ray Crystal Structure of *2(**1,2-di-I-tFb**)∙(**ht-2,4-tpcb**)*

**Figure 2:**
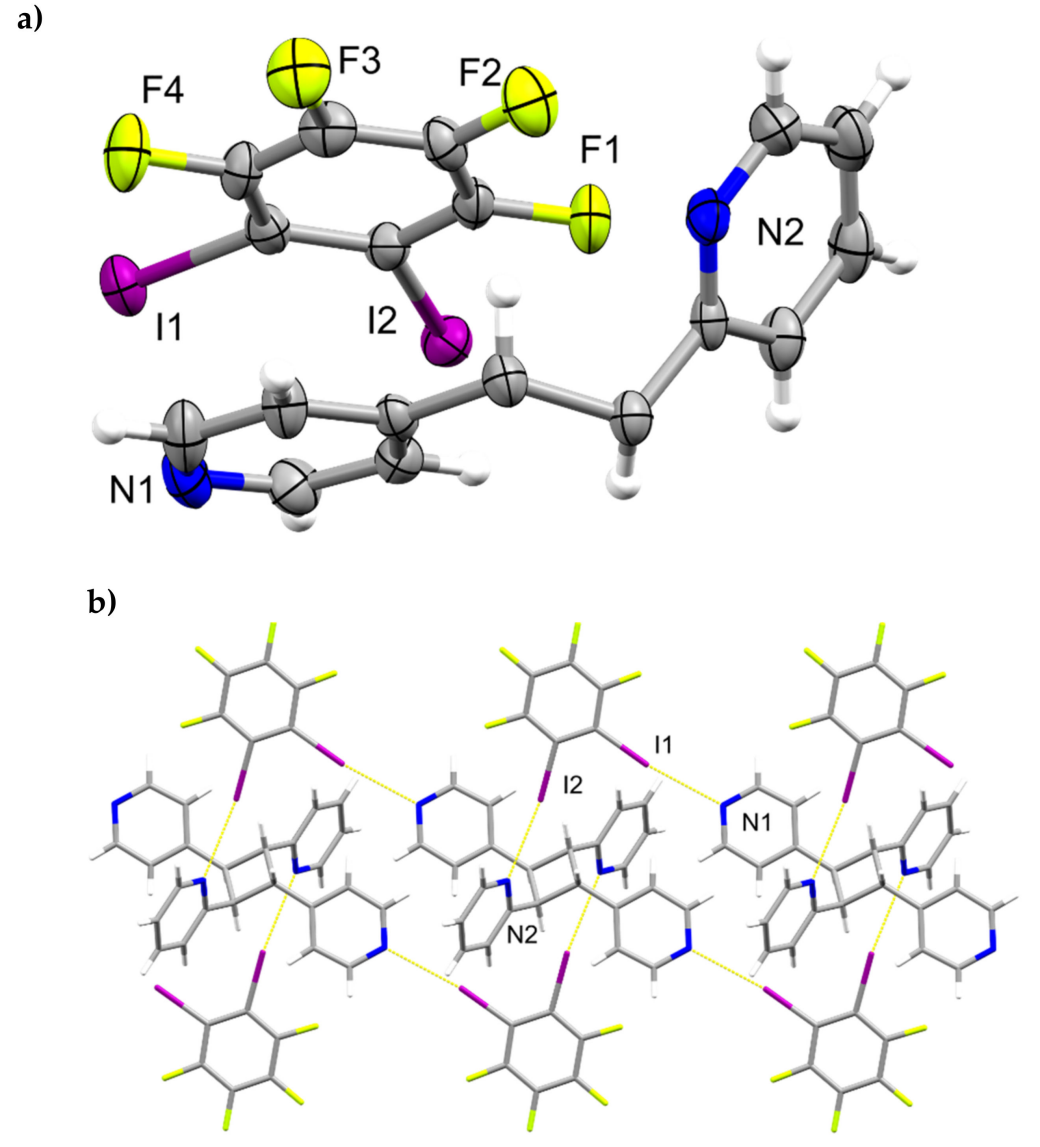

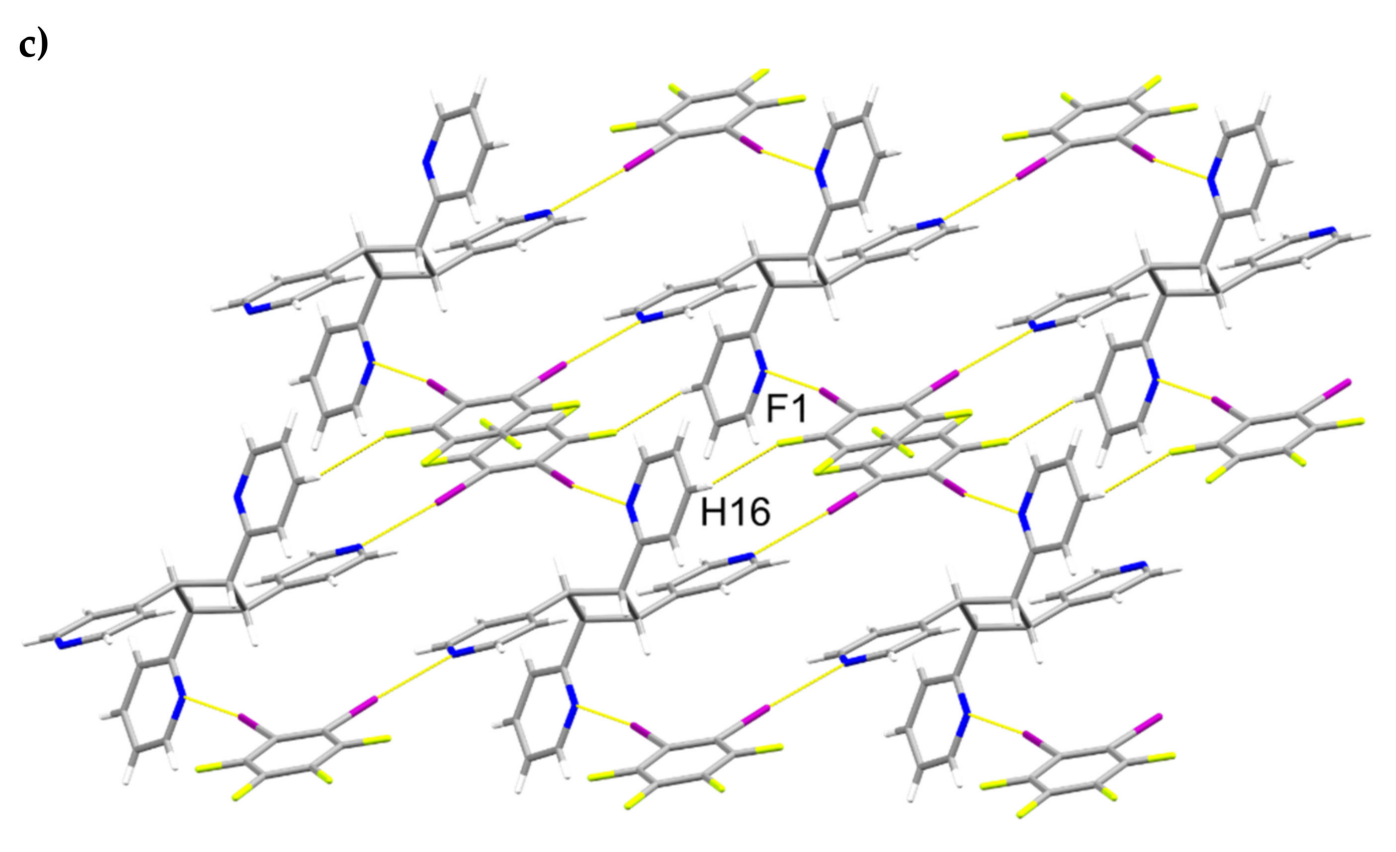
Perspectives of 2(**1,2-di-I-tFb**)∙(***ht*-2,4-tpcb**): (**a**) asymmetric unit (anisotropic displacement ellipsoids at 50% probability); (**b**) 1D assemblies; and (**c**) sheets based on C-H∙∙∙F forces.

A single-crystal X-ray structure determination confirmed the *ht*-regiochemistry of ***ht*-2,4-tpcb** (Figure 2). The components of 2(**1,2-di-I-tFb**)∙(***ht*-2,4-tpcb**) crystallize in the triclinic space group *P*$\bar{1}$ (Table 1). The asymmetric unit consists of one full molecule of **1,2-di-I-tFb** and one half-molecule of ***ht*-2,4-tpcb**, with the cyclobutane located around a crystallographic center of inversion (Figure 2a). The 2-pyr rings of ***hh*-2,4-tpcb** adopt an *anti*-conformation (Figure 2b). The components assemble primarily via N∙∙∙I X-bonds (*d*(N1∙∙∙I1) = 2.946(4) Å; *d*(N2∙∙∙I2) = 3.022(4) Å, Table 3) to form 1D assemblies that propagate along the crystallographic *c*-axis with **1,2-di-I-tFb** bridging ***ht*-2,4-tpcb** (Figure 2b, Table 4). Adjacent assemblies interact primarily via edge-to-edge C-H∙∙∙F forces (*d*(H16∙∙∙F1) ~ 2.52 Å) to form 2D sheets (Figure 2c,d, Table 4).

### 2.3. X-ray Crystal Structure of *(**1,3-di-I-tFb**)∙(**2,4-bpe**)*

**Figure 3:**
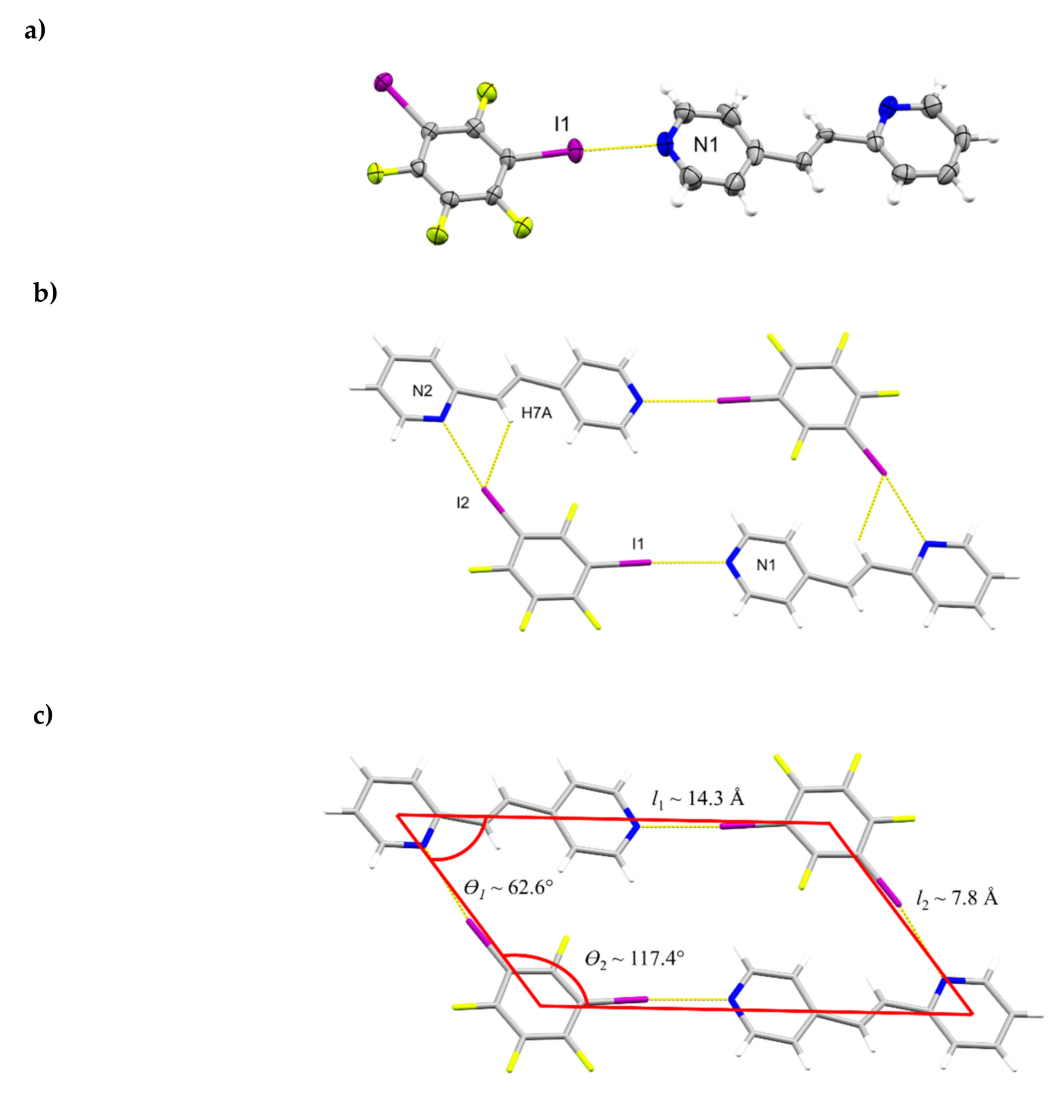

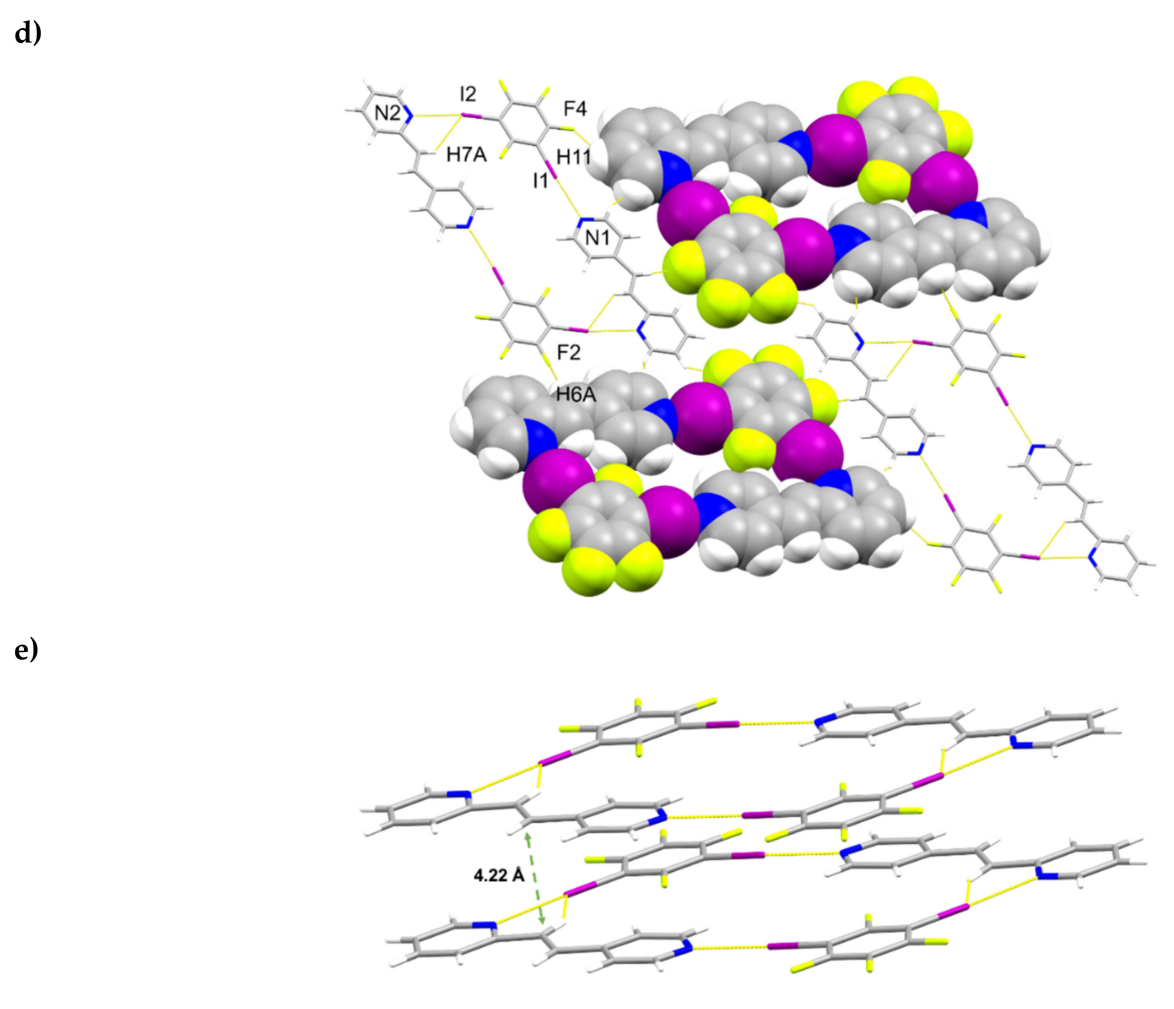
Perspectives of (**1,3-di-I-tFb**)∙(**2,4-bpe**) (minor fraction of alkene disorder omitted for clarity): (**a**) asymmetric unit (anisotropic displacement ellipsoids at 50% probability); (**b**) four-component assembly; (**c**) rhomboidal repeat highlighted (C-H∙∙∙I contacts omitted for clarity); (**d**) sheets with two assemblies shown as space filling; and (**e**) nearest-neighbor alkenes between adjacent sheets highlighting closest alkene separation (green dashed arrow).

The components of (**1,3-di-I-tFb**)∙(**2,4-bpe**) crystallize in the monoclinic space group *P*2_1_/*n* (Figure 3, Table 5). The asymmetric unit consists of one full molecule each of **1,3-di-I-tFb** and **2,4-bpe** (Figure 3a). The alkene C=C bond of **2,4-bpe** lies disordered over two sites (occupancies: 0.75/0.25). The pyr rings of **2,4-bpe** lie approximately coplanar (*ϕ* ~ 3.9°, Table 2). The components assemble via a combination of N∙∙∙I X-bonds (*d*(N1∙∙∙I1) = 2.795(4) Å); *d*(N2∙∙∙I2) = 2.926(4) Å, Table 3) and offset, edge-to-edge C-H∙∙∙F forces (*d*(H7A∙∙∙I2) ~ 2.99 Å) to form discrete, four-component rhomboids (*ϴ*_1_ ~ 62.6°; *ϴ*_2_ ~117.4°; *l*_1_ ~ 14.3 Å; *l*_2_ ~ 7.8 Å) (Figure 3b,c, Table 4). Adjacent assemblies interact primarily via edge-to-edge C-H∙∙∙F forces between an alkenyl H-atom of **2,4-bpe** and **1,3-di-I-tFb** (*d*(H6A∙∙∙F2) ~2.54 Å; *d*(H11∙∙∙F4) ~ 2.53 Å) to form sheets (Figure 3d, Table 4). Adjacent sheets interact via offset, face-to-face *π*-stacks between the 2-pyr and 4-pyr rings of neighboring molecules of **2,4-bpe** (*d*(pyr_N1_∙∙∙pyr_N2_) ~ 5.10 Å). Nearest-neighbor molecules of **2,4-bpe** stack head-to-head (*hh*) with alkene C=C bonds separated by 4.22 Å and with closest alkene C=C bonds stacked in a combination of parallel and crisscrossed geometries (Figure 3d) [18,19].

The alkene C=C bonds of (**1,3-di-I-tFb**)∙(**2,4-bpe**) are photoactive. When (**1,3-di-I-tFb**)∙(**2,4-bpe**) was subjected to UV-irradiation (broadband Hg lamp, 80 h), **2,4’-bpe** underwent a photodimerization to generate ***hh*-2,4-tpcb** stereospecifically, regiospecifically, and in quantitative conversion [20] (Scheme 2, Table 4). The formation of the photoproduct was evidenced by the complete disappearance of the pair of olefinic doublets (*δ*_H_ = 7.65, 7.59 ppm) with concomitant emergence of a pair of cyclobutane resonances (*δ*_H_ = 4.89, 4.69 ppm) in the ^1^H NMR spectrum (Figure S3 of the ESI). The reactivity was presumably supported by the pedal-like motion of the disordered alkene C=C bonds upon irradiation [19,21,22,23,24,25,26,27,28,29,30]. 

### 2.4. X-ray Crystal Structure of *2(**1,3-di-I-tFb**)∙(**hh-2,4-tpcb**)*

**Figure 4:**
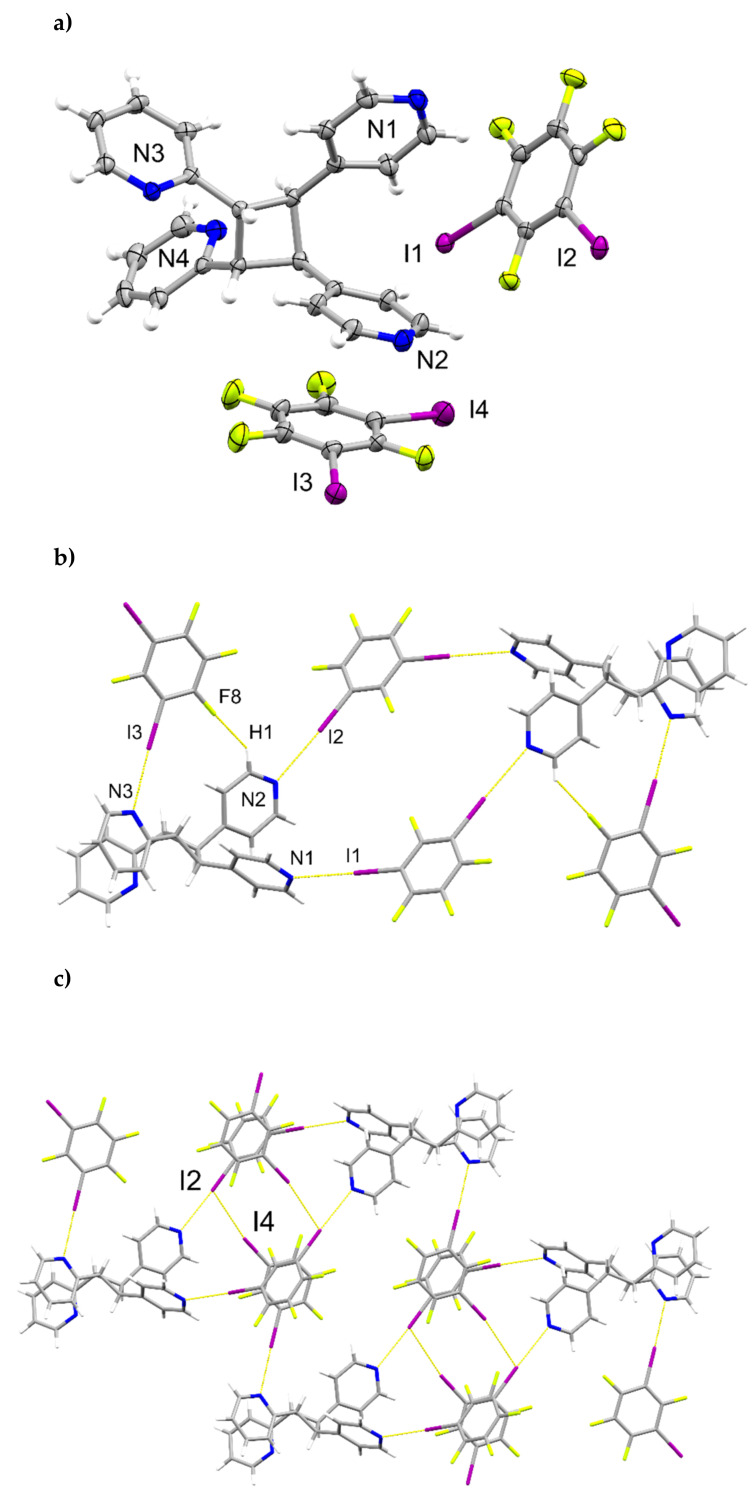
Perspectives of 2(**1,3-di-I-tFb**)∙(***hh*-2,4-tpcb**): (**a**) asymmetric unit (thermal ellipsoids shown at 50% probability); (**b**) discrete, six-component assembly; and (**c**) adjacent assemblies illustrating Type II I∙∙∙I X-bonds.

A single-crystal X-ray structure determination confirmed the *hh*-regiochemistry of ***hh*-2,4-tpcb** (Figure 4). The components of 2(**1,3-di-I-tFb**)∙(***hh*-2,4-tpcb**) crystallize in the triclinic space group *P*$\bar{1}$ (Table 5). The asymmetric unit consists of one full molecule of ***hh*-2,4-tpcb** and two full molecules of **1,3-di-I-tFb** (Figure 4a). The 2-pyr rings of ***hh*-2,4-tpcb** adopt an *anti*-conformation. The components assemble primarily via N∙∙∙I X-bonds (*d*(N1∙∙∙I1) = 2.826(2) Å; *d*(N2∙∙∙I2) = 2.892(2) Å; *d*(N3∙∙∙I3) = 2.826(2) Å; Table 3) and secondary edge-to-edge C-H∙∙∙F forces (*d*(H1∙∙∙F8) ~ 2.55 Å) to form discrete, six-component assemblies (Figure 4b, Table 4). In contrast to 2(**1,2-di-I-tFb**)∙(***ht*-2,4-tpcb**), wherein all four pyr N-atoms participate in N∙∙∙I X-bonds, only three N-atoms of 2(**1,3-di-I-tFb**)∙(***hh*-2,4-tpcb**) participate in N∙∙∙I X-bonds (Figure 4b). Adjacent assemblies interact primarily via Type II [22] I∙∙∙I X-bonds (*d*(I2∙∙∙I4) = 3.6970(4) Å) to form an extended X-bonded network (Figure 4c, Table 4). 

### 2.5. X-ray Crystal Structure of *(**1,4-di-I-tFb**)∙(**2,4-bpe**)*

**Figure 5:**
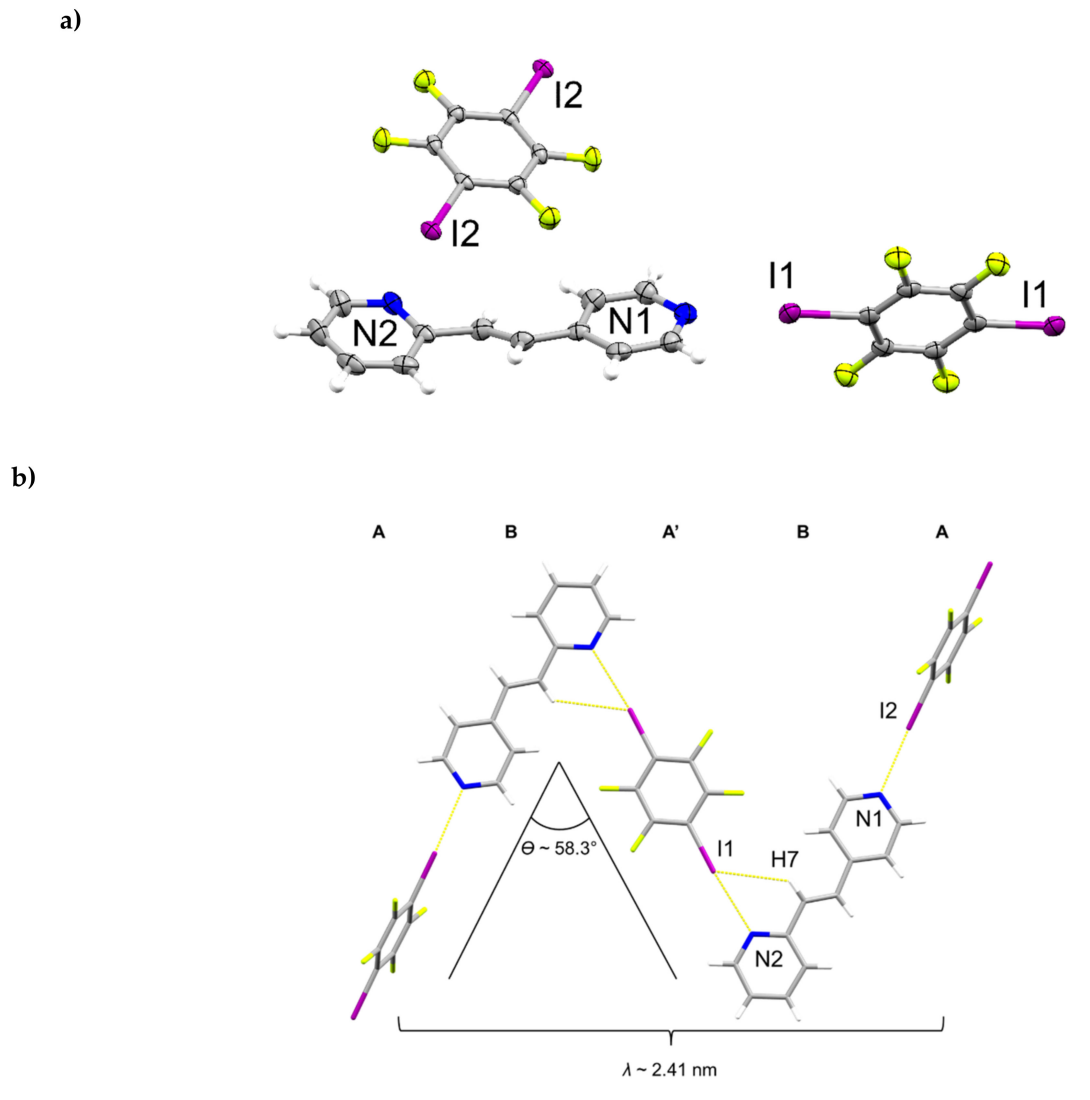

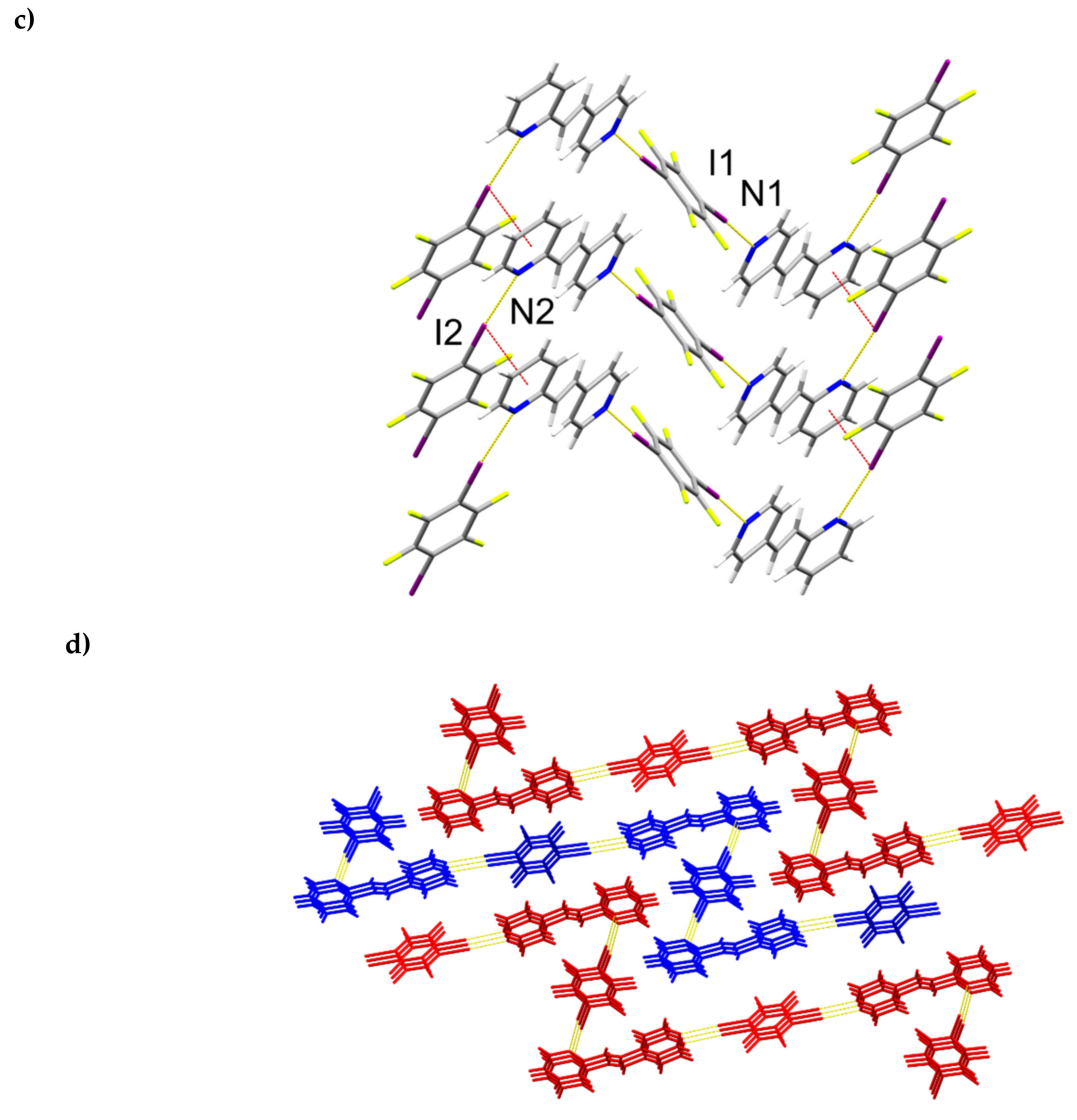
Perspectives of (**1,4-di-I-tFb**)∙(**2,4-bpe**): (**a**) ORTEP (thermal ellipsoids shown at 50% probability; view along *b*); (**b**) zig-zag chains illustrating ABA’B repeat; (**c**) columns highlighting Type II *π*∙∙∙I X-bonds (red dashed lines) (C-H∙∙∙I contacts omitted for clarity; view along *c*); and (**d**) herringbone packing of columns.

The components of (**1,4-di-I-tFb**)∙(**2,4-bpe**) crystallize in the monoclinic space group *P*2_1_/*c* (Figure 5, Table 5). The asymmetric unit consists of one full molecule of **2,4-bpe** and two crystallographically unique half-molecules of **1,2-di-I-tFb**, the rings of which are both located around crystallographic centers of inversion (Figure 5a). The pyr rings of **2,4-bpe** lie slightly twisted from coplanarity (*ϕ* ~ 9.9°, Table 2). The components assemble via a combination of N∙∙∙I X-bonds (*d*(N1∙∙∙I2) = 2.802(4) Å; *d*(N2∙∙∙I1) = 2.884(3) Å, Table 3) and offset, edge-to-edge C-H∙∙∙I forces (*d*(H7∙∙∙I1) ~ 3.06 Å) to form infinite zig-zag chains (*λ* ~ 2.41 nm; *ϴ* ~ 58.3°) characterized by an ABA’B repeat (Figure 5b, Table 4). Adjacent chains run parallel and interact primarily via Type II *π*∙∙∙I X-bonds (*d*(pyr_N2_∙∙∙I2) ~ 3.70 Å) to form columns (Figure 5c, Table 4). Between adjacent chains, nearest-neighbor alkenes stack parallel, appreciably offset, and separated by 5.22 Å (Figure 5c), a geometry that does not satisfy the criteria of Schmidt [16] for topochemical [2+2] photodimerization. The X-bonded columns run antiparallel and pack in a herringbone pattern (Figure 5d). UV-irradiation (broadband Hg lamp, 20 h) of a sample of (**1,4-di-I-tFb**)∙(**2,4-bpe**) revealed the solid to be photostable (Table 4).

### 2.6. Structural Considerations

The unsymmetrical nature of **2,4-bpe** provides two different pyr N-atoms (i.e., 2-pyr versus 4-pyr) to participate in X-bonding. We note that in virtually all cases, the N∙∙∙I X-bond lengths involving I-atoms of the X-bond donors **1,*n*-di-I-tFb** to N-atoms of the X-bond acceptors **2,4-bpe**, ***ht*-2,4-tpcb**, and ***hh*-2,4-tpcb** are shorter for 4-pyr versus 2-pyr (Table 3). The average percent relative shortening (prs) values for N∙∙∙I X-bonds to 2-pyr versus 4-pyr N atoms were 15.7% and 19.5%, respectively (Table 3). Given that p*K*_a_ values for similar 4-pyr and 2-pyr analogs are comparable [31], we attribute the observation to greater steric crowding between the lone pair on the N-atom of 2-pyr versus 4-pyr rings. Crowding would presumably preclude maximal orbital overlap (i.e., strongest X-bond formation) between the N-atom lone pair and the *σ*-hole of the relatively large I-atoms relative to an appreciably less congested 4-pyr N-atom.

## 3. Materials and Methods

### 3.1. General Experimental

All reagents and solvents (synthesis grade) were purchased from commercial sources and used as received unless otherwise stated. 1,2-diiodotetrafluorobenzene (**1,2-di-I-tFb**; 99%), *trans*-1-(2-pyridyl)-2-(4-pyridyl)ethylene (**2,4-bpe**; 97%), and 1,4-diiodotetrafluorobenzene (**1,4-di-I-tFb**, 98%) were purchased from Aldrich^©^. 1,3-diiodotetrafluorobenzene (**1,3-di-I-tFb**; 97%) was purchased from Apollo Scientific^©^ (Bredbury, UK). Chloroform (CHCl_3_; certified ACS grade, ≥99.8%, approximately 0.75% EtOH as preservative) was purchased from Fisher Chemical^©^ (Hampton, NH, USA). All cocrystal syntheses were conducted in screw-cap glass scintillation vials. For cocrystal syntheses, “thermal dissolution” refers to the process of: (1) combining both cocrystal components in the same screw-cap glass vial; (2) adding solvent portion-wise while maintaining a saturated mixture at rt; and (3) tightly capping the vial and heating the mixture on a hotplate until all solids dissolve to afford a homogeneous solution in the minimum necessary volume of solvent. Compositions of all single crystals were shown to be representative of the bulk material by matching experimental pXRD patterns with those simulated from scXRD data. Photoreactions were conducted in an ACE^®^ photo cabinet equipped with a water-cooled ACE^®^ quartz, 450 W, broadband (*λ* = 1367.3–222.4 nm), medium pressure, Hg-vapor lamp (of the total energy emitted by the broadband lamp, approximately 40–48% is in the ultraviolet portion of the spectrum, 40–43% in the visible, and the balance in the infrared). Photoreactions were conducted by: (1) grinding single crystals of the cocrystal to a fine powder with an agate mortar and pestle; (2) smearing the powder between two UV-transparent Pyrex^®^ plates to create the thinnest layer possible; and (3) irradiating the powder in 10 h intervals, taking care to ensure uniform irradiation. Uniform irradiation of the powdered cocrystals was accomplished by: (1) occasionally (between every other irradiation interval) scraping (razor blade) the irradiated powder from both plates of the plate assembly; (2) combining the powder from both plates; (3) homogenizing the combined, bulk powder via thorough grinding (agate mortar and pestle); and (4) redistributing the homogenized powder between both plates. The plate assembly was also flipped between irradiation intervals to ensure equal irradiation of both faces of the plate assembly.

### 3.2. Synthetic Procedures

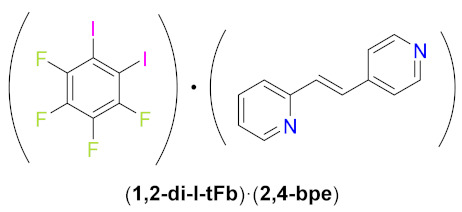



(**1,2-di-I-tFb**)∙(**2,4-bpe**). Cocrystals of (**1,2-di-I-tFb**)∙(**2,4-bpe**) were obtained by thermal dissolution of **2,4-bpe** (192.9 mg, 1.03 mmol) and **1,2-di-I-tFb** (418.7 mg, 1.03 mmol, 1.0 equiv) in CHCl_3_ (7.0 mL). Upon cooling to rt, single crystals of (**1,2-di-I-tFb**)∙(**2,4-bpe**)—colorless laths, suitable for scXRD—formed within 15 d.

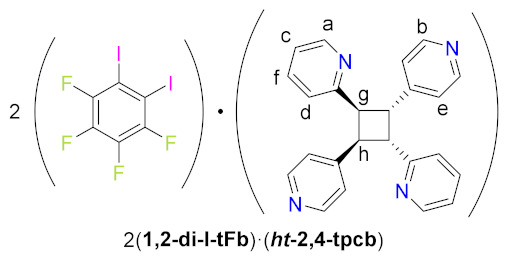



**2**(**1,2-di-I-tFb**)∙(***ht*-2,4-tpcb**). Single crystals of (**1,2-di-I-tFb**)∙(**2,4-bpe**) were ground to a fine powder using an agate mortar and pestle and smeared between two Pyrex^®^ plates. The plate assembly was placed in an ACE^®^ photo cabinet. After 100 h, ^1^H NMR assay revealed quantitative, stereospecific, and regiospecific conversion to 2(**1,2-di-I-tFb**)·(***ht*-2,4-tpcb**). The product powder was scraped from the plates, dissolved in the minimum volume of boiling CHCl_3_, and allowed to slowly cool to rt: single crystals of 2(**1,2-di-I-tFb**)·(***ht*-2,4-tpcb**)—colorless, irregular prisms, suitable for scXRD—formed within 6 d. Analytical data: **^1^H NMR** (400 MHz, DMSO-*d*_6_): *δ* 8.40 (dd, *J* = 4.8 0.8 Hz, 2H_a_), 8.27 (dd, *J* = 4.6, 1.4 Hz, 4H_b_), 7.55 (app td, *J* = 7.6, 1.8 Hz, 2H_c_), 7.19 (d, *J* = 7.8 Hz, 2H_d_), 7.16 (d, *J* = 6.0 Hz, 4H_e_), 7.06 (ddd, *J* = 7.5, 4.9, 0.9 Hz, 2H_f_), 4.87-4.82 (m, 2H_g_), 4.79-4.74 (m, 2H_h_). Spectral data were consistent with those previously reported [17] for the same compound.

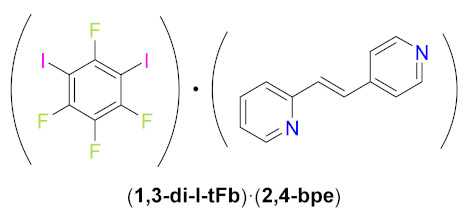



(**1,3-di-I-tFb**)∙(**2,4-bpe**). Cocrystals of (**1,3-di-I-tFb**)∙(**2,4-bpe**) were obtained by thermal dissolution of **2,4-bpe** (191.1 mg, 1.02 mmol) and **1,3-di-I-tFb** (430.5 mg, 1.02 mmol, 1.0 equiv) in CHCl_3_ (7.0 mL). Upon cooling to rt, single crystals of (**1,3-di-I-tFb**)∙(**2,4-bpe**)—colorless plates, suitable for scXRD—formed within 15 d.

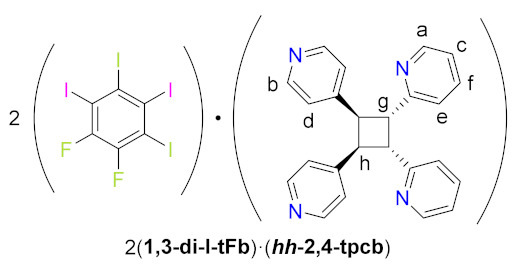



**2(1,3-di-I-tFb**)**∙(*hh*-2,4-tpcb**). Single crystals of (**1,3-di-I-tFb**)∙(**2,4-bpe**) were ground to a fine powder using an agate mortar and pestle and smeared between two Pyrex^®^ plates. The plate assembly was placed in an ACE^®^ photo cabinet. After 80 h, ^1^H NMR assay revealed quantitative, stereospecific, and regiospecific conversion to 2(**1,3-di-I-tFb**)·(***hh*-2,4-tpcb**). The product powder was scraped from the plates, dissolved in the minimum volume of boiling CHCl_3_, and allowed to slowly cool to rt: single crystals of 2(**1,3-di-I-tFb**)·(***hh*-2,4-tpcb**)—colorless prisms, suitable for scXRD—formed within 6 d. Note: When preparing a sample of these crystals for pXRD assay (dry-grinding with an agate mortar and pestle), the solid initially assumed a moist, paste-like consistency, but eventually dried upon sitting exposed to air for several hours at rt. Analytical data: **^1^H NMR** (400 MHz, DMSO-*d*_6_): *δ* 8.33 (ddd, *J* = 4.8, 1.7, 0.9 Hz, 2H_a_), 8.31 (dd, *J* = 4.5, 1.5 Hz, 4H_b_), 7.50 (app td, *J* = 7.7, 1.8 Hz, 2H_c_), 7.21 (dd, *J* = 4.5, 1.6 Hz, 4H_d_), 7.12 (d, *J* = 7.8 Hz, 2H_e_), 7.02 (ddd, *J* = 7.5, 4.9, 1.0 Hz, 2H_f_), 4.89 (d, *J* = 6.4 Hz, 2H_g_), 4.69 (d, *J* = 6.3 Hz, 2H_h_). Spectral data were consistent with those previously reported [20] for the same compound.

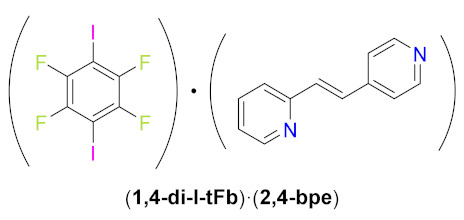



(**1,4-di-I-tFb**)**∙**(**2,4-bpe**)**.** Cocrystals of (**1,4-di-I-tFb**)∙(**2,4-bpe**) were obtained by thermal dissolution of **2,4-bpe** (194.0 mg, 1.03 mmol) and **1,4-di-I-tFb** (428.8 mg, 1.05 mmol, 1.0 equiv) in CHCl_3_ (7.0 mL). Upon cooling to rt, single crystals of (**1,4-di-I-tFb**)∙(**2,4-bpe**)—colorless blades, suitable for scXRD—formed within 11 d.

### 3.3. H NMR Spectroscopy

Proton nuclear magnetic resonance (^1^H NMR) spectra were recorded at room temperature on a Bruker^®^ AVANCE NEO-400 spectrometer (Bruker Corp., Billerica, MA, USA) operating at 400 MHz using a liquid-N_2_-cooled double-resonance broadband Prodigy^TM^ cryoprobe. ^1^H NMR data are reported as follows: chemical shift (*δ*, ppm), multiplicity (d = doublet, dd = doublet of doublets, ddd = doublet of doublet of doublets, app td = apparent triplet of doublets, m = multiplet), coupling constant(s) (*J*, Hz), and integration. Chemical shift values were calibrated relative to residual solvent resonance (central peak of DMSO: *δ*_H_ = 2.50 ppm) as the internal standard. All ^1^H NMR data were collected and plotted within the Bruker^®^ TopSpin^TM^ v3.6.1 software suite.

### 3.4. Powder X-ray Diffraction (pXRD)

Powder X-ray diffraction (pXRD) data were collected at room temperature on a Bruker^®^ D8 Advance X-ray diffractometer (Bruker Corp., Billerica, MA, USA) on samples mounted on glass slides. Each sample was finely ground using an agate mortar and pestle prior to mounting. Instrument parameters: radiation wavelength, CuK*α* (*λ* = 1.5418 Å); scan type, coupled TwoTheta/Theta; scan mode, continuous PSD fast; scan range, 5–40° two-theta; step size, 0.02°; voltage, 40 kV; current, 30 mA. Background subtractions were applied to all experimentally collected data within the Bruker^®^ DIFFRAC.EVA v3.1 software suite. All data were plotted in the Microsoft^®^ Excel 2016 software suite. Simulated pXRD patterns were calculated from scXRD data within the CCDC Mercury [32] software suite.

### 3.5. Single-Crystal X-ray Diffraction (scXRD)

Single-crystal X-ray diffraction data were collected on a Bruker^®^ D8 VENTURE^®^ (DUO) CCD diffractometer (Bruker Corp., Billerica, MA, USA) equipped with a Bruker^®^ PHOTON III^®^ photon counting detector and an Oxford Cryostream^®^ 800 series cold N_2_ gas stream cooling system (Oxford Cryosystems, Oxford, UK). Data were collected at a low temperature (150(2) K) using graphite-monochromated MoK*α* radiation (*λ* = 0.71073 Å). Crystals were mounted in Paratone^®^ oil on a MiTeGen^©^ magnetic mount. Data collection strategies for ensuring maximum data redundancy and completeness were calculated using the Bruker^®^ Apex II^TM^ software suite. Data collection, initial indexing, frame integration, Lorentz-polarization corrections and final cell parameter calculations were likewise accomplished using the Apex II software suite. Multi-scan absorption corrections were performed using SADABS [33]. Structure solution and refinement were accomplished using SHELXT [34] and SHELXL [35], respectively, within the Olex2 [36] v1.2 graphical user interface. Space groups were unambiguously verified using the PLATON^©^ [37] executable. All non-hydrogen atoms were refined anisotropically. All hydrogen atoms were attached via a riding model at calculated positions using suitable HFIX commands. The occupancies of the major and minor positions for the disordered alkene C=C core within (**1,3-di-I-tFb**)·(**2,4-bpe**) converged to their respective ratios after each was identified in the difference map and freely refined. Figures of all structures were rendered in the CCDC Mercury [32] software suite.

## 4. Conclusions

N∙∙∙I X-bonds have been used to support topochemical [2+2] photodimerizations of an unsymmetrical alkene to generate either of two regioisomeric cyclobutane photoproducts in the organic solid state. The transformations proceeded stereospecifically, regiospecifically, and quantitatively to generate ***ht*-** or ***hh*-2,4-tpcb**. Our contribution, thus, can be considered to afford à la carte access to either regioisomer. The formation of each product is achieved from the same alkene substrate, **2,4’-bpe**, using commercially available X-bond donor cocrystal formers. Our future efforts will aim to expand the scope of the supramolecular methodology described herein to other unsymmetrical alkenes to afford access to additional unsymmetrical cyclobutane photoproducts.

## Figures and Tables

**Table 1:** Crystallographic data and structure refinement statistics *.

Cocrystal	(1,2-di-I-tFb)·(2,4-bpe)	2(1,2-di-I-tFb)·(*ht*-2,4-tpcb)
CCDC deposition number	2104746	2114529
Empirical formula	C_18_H_10_F_4_I_2_N_2_	C_36_H_20_F_8_I_4_N_4_
Formula weight/g·mol^−1^	584.08	1168.16
Temperature/K	150(2)	150(2)
Crystal system	Triclinic	Triclinic
Space group	*P*-1	*P*-1
*a*/Å	7.5879(8)	9.1499(10)
*b*/Å	13.1862(13)	10.6011(11)
*c*/Å	19.2918(19)	10.9421(12)
*α*/°	80.092(5)	62.054(5)
*β*/°	89.530(5)	77.014(5)
*γ*/°	74.125(5)	76.828(5)
Volume/Å^3^	1827.4(3)	904.32(17)
Z	4	1
*ρ*_calc_/g·cm^−3^	2.12	2.15
*μ*/mm^−1^	3.484	3.521
*F*(000)	1096	548
Crystal size/mm^3^	0.11 × 0.06 × 0.03	0.10 × 0.09 × 0.06
Radiation	MoK*α* (*λ* = 0.71073)	MoK*α* (*λ* = 0.71073)
2*Θ* range for data collection/°	4.21 to 50.25	4.25 –to 50.25
Index ranges	−9 ≤ h ≤ 9, −15 ≤ k ≤ 15, −23 ≤ l ≤ 21	−10 ≤ h ≤ 10, −12 ≤ k ≤ 12, −13 ≤ l ≤ 13
Reflections collected	43105	40307
Independent reflections	6502 [*R*_int_ = 0.0349, *R*_sigma_ = 0.0229]	3220 [*R*_int_ = 0.0830, *R*_sigma_ = 0.0312]
Data/restraints/parameters	6502/0/549	3220/0/235
Goodness-of-fit on *F*^2^	1.066	1.060
Final *R* indices [*I* ≥ 2*σ* (*I*)]	*R*_1_ = 0.0170	*R*_1_ = 0.0358
	*wR*_2_ = 0.0363	*wR*_2_ = 0.0781
*R* indices (all data)	*R*_1_ = 0.0211	*R*_1_ = 0.0377
	*wR*_2_ = 0.0379	*wR*_2_ = 0.0790
Largest diff. peak/hole/e·Å^−3^	0.66/−0.36	2.44/−0.94

* $R_{1}=\sum \left|\left|F_{o}\right|\;–\;\left|F_{c}\right|\right|/\sum \left|F_{o}\right|,\;$ $wR_{2}=\left[\sum w{\left(F_{o}^{2}\;–\;F_{c}^{2}\right)}^{2}\right]/\sum {\left[w{\left(F_{o}^{2}\right)}^{2}\right]}^{1/2}$. Goodness-of-fit on F^2^ $={\left[\sum w{\left(\left|F_{o}\right|-\left|F_{c}\right|\right)}^{2}/\left(N_{\mathrm{obs}}-N_{\mathrm{parameter}}\right)\right]}^{1/2}$.

**Table 2:** Twist angles (*ϕ*) of pyr rings of **2,4-bpe** in binary cocrystals.

Crystal	2,4-bpe Molecule	Twist Angle *ϕ*/°
(**1,2-di-I-tFb**)·(**2,4-bpe**)	N1/N2	3.4
	N3/N4	8.0
(**1,3-di-I-tFb**)·(**2,4-bpe**)	N1/N2	3.8
(**1,4-di-I-tFb**)·(**2,4-bpe**)	N1/N2	9.9

**Table 3:** X-bond metrics for cocrystals.

Cocrystal	X-Bond	Pyridyl Substitution	*d*(N···I)/Å	*Θ*(C-I···N)/°	X-Bond Type	prs *
**(1,2-di-I-tFb)·(2,4-bpe)**	I1···N1	4	2.808(2)	176.6	I	20
	I2···N4	2	3.054(2)	174.0	I	13
	I3···N3	4	2.814(2)	176.8	I	20
	I4···N2	2	3.147(2)	171.9	I	11
2(**1,2-di-I-tFb)·(*ht*-2,4-tpcb)**	I1···N1	4	2.946(4)	172.6	I	17
	I2···N2	2	3.022(4)	172.1	I	14
**(1,3-di-I-tFb)·(2,4-bpe)**	I1···N1	4	2.795(4)	177.2	I	21
	I2···N2	2	2.926(4)	171.8	I	17
2**(1,3-di-I-tFb)·(*hh*-2,4-tpcb)**	I1···N1	4	2.826(2)	174.4	I	20
	I2···N2	4	2.892(2)	177.9	I	18
	I3···N3	2	2.826(2)	169.0	I	20
**(1,4-di-I-tFb)·(2,4-bpe)**	I2···N1	4	2.802(4)	177.5	I	21
	I1···N2	2	2.884(3)	175.6	I	18

* prs ≡ percent relative shortening = {1 − *d*(N···I)/[*r*_vdW_(I) + *r*_vdW_(N)]}·100, where *r*_vdW_(N) and *r*_vdW_(I) are the van der Waals radii for nitrogen and iodine, respectively, with *r*_vdW_(N) = 1.55 Å and *r*_vdW_(I) = 1.98 Å.

**Table 4:** Structural features of cocrystals.

Cocrystal	Primary Assembly	Secondary Assembly	Photoreactivity
**(1,2-di-I-tFb)·(2,4-bpe)** (2104746)	infinite 1D tape based on N∙∙∙I	infinite 2D corrugated layers	active
2**(1,2-di-I-tFb)·(*rctt*-*ht*-2,4-tpcb)** (2114529)	infinite 1D chain based on N∙∙∙I	infinite sheets based on C-H∙∙∙F	-
(**1,3-di-I-tFb**)·(**2,4-bpe**) (2104747)	discrete, four-component assembly based on N∙∙∙I	infinite sheets based on C-H∙∙∙F	active
2**(1,3-di-I-tFb)·(*rctt*-*hh*-2,4-tpcb)** (2114285)	discrete, six-component assembly based on N∙∙∙I and C-H∙∙∙F	3D network based on Type II I∙∙∙I	-
(**1,4-di-I-tFb**)·(**2,4-bpe**) (2104748)	infinite zig-zag chains based on N∙∙∙I	infinite columns based on Type II I∙∙∙I	inert

**Table 5:** Crystallographic data and structure refinement statistics *.

Cocrystal	(1,3-di-I-tFb)·(2,4-bpe)	2(1,3-di-I-tFb)·(*hh*-2,4-tpcb)	(1,4-di-I-tFb)·(2,4-bpe)
CCDC deposition number	2104747	2114285	2104748
Empirical formula	C_18_H_10_F_4_I_2_N_2_	C_36_H_20_F_8_I_4_N_4_	C_18_H_10_F_4_I_2_N_2_
Formula weight/g·mol^−1^	584.08	1168.16	584.08
Temperature/K	150(2)	150(2)	150(2)
Crystal system	Monoclinic	Triclinic	Monoclinic
Space group	*P*2_1_/*n*	*P-1*	*P*2_1_/*c*
*a*/Å	4.2177(4)	8.1884(6)	17.7168(18)
*b*/Å	27.254(3)	14.2749(14)	5.2208(5)
*c*/Å	16.1797(16)	16.3737(16)	20.159(2)
*α*/°	90	82.111(5)	90
*β*/°	90.940(5)	87.336(5)	103.519(5)
*γ*/°	90	73.759(5)	90
Volume/Å^3^	1859.6(3)	1820.1(3)	1813.0(3)
Z	4	2	4
*ρ*_calc_/g·cm^−3^	2.09	2.13	2.14
*μ*/mm^−1^	3.424	3.498	3.512
*F*(000)	1096	1096	1096
Crystal size/mm^3^	0.15 × 0.15 × 0.05	0.07 × 0.06 × 0.05	0.27 × 0.09 × 0.02
Radiation	MoK*α* (*λ* = 0.71073)	MoK*α* (*λ* = 0.71073)	MoK*α* (*λ* = 0.71073)
2*Θ* range for data collection/°	5.036 to 50.244	4.152 to 50.246	4.156 to 50.246
Index ranges	−5 ≤ h ≤ 5, −32 ≤ k ≤ 32, −18 ≤ l ≤ 19	−9 ≤ h ≤ 9, −17 ≤ k ≤ 17, −18 ≤ l ≤ 19	−21 ≤ h ≤ 21, −24 ≤ k ≤ 24, −23 ≤ l ≤ 23
Reflections collected	38879	34954	46454
Independent reflections	3283 [*R*_int_ = 0.0379, *R*_sigma_ = 0.0213]	6475 [*R*_int_ = 0.0264, *R*_sigma_ = 0.0184]	3233 [*R*_int_ = 0.0362, *R*_sigma_ = 0.0153]
Data/restraints/parameters	3234/18/253	6475/0/469	3233/0/235
Goodness-of-fit on *F*^2^	1.082	1.033	1.049
Final *R* indices [*I* ≥ 2*σ* (*I*)]	*R*_1_ = 0.0299	*R*_1_ = 0.0174	*R*_1_ = 0.0248
	*wR*_2_ = 0.0802	*wR*_2_ = 0.0365	*wR*_2_ = 0.0538
*R* indices (all data)	*R*_1_ = 0.0306	*R*_1_ = 0.0222	*R*_1_ = 0.0271
	*wR*_2_ = 0.0807	*wR*_2_ = 0.0387	*wR*_2_ = 0.0551
Largest diff. peak/hole/e·Å^−3^	0.79/−0.63	1.20/−1.00	0.71/−0.60

* $R_{1}=\sum \left|\left|F_{o}\right|-\left|F_{c}\right|\right|/\sum \left|F_{o}\right|,\;$ $wR_{2}=\left[\sum w{\left(F_{o}^{2}-F_{c}^{2}\right)}^{2}\right]/\sum {\left[w{\left(F_{o}^{2}\right)}^{2}\right]}^{1/2}$. Goodness-of-fit on *F*^2^ $=\;\;{\left[\sum w{\left(\left|F_{o}\right|-\left|F_{c}\right|\right)}^{2}/\left(N_{\mathrm{obs}}-N_{\mathrm{parameter}}\right)\right]}^{1/2}$.

## Data Availability

The data presented in this study are available in the article, Cambridge Structural Database (CSD), or the Supplementary Materials.

## Supplementary Materials

The following are available online. Figure S1: Stacked ^1^H NMR spectra of (**1,2-di-I-tFb**)∙(**2,4-bpe**) and 2(**1,2-di-I-tFb**)∙(***ht*-2,4-tpcb)**; Figure S2: ^1^H NMR spectrum of 2(**1,2-di-I-tFb**)∙(***ht*-2,4-tpcb**); Figure S3: Stacked ^1^H NMR spectra of (**1,3-di-I-tFb**)∙(**2,4-bpe**) and 2(**1,3-di-I-tFb**)∙(***hh*-2,4-tpcb**); Figure S4: ^1^H NMR spectrum of 2(**1,3-di-I-tFb**)∙(***hh*-2,4-tpcb**); Figure S5: pXRD pattern of (**1,2-di-I-tFb**)·(**2,4-bpe**); Figure S6: pXRD pattern of 2(**1,2-di-I-tFb**)·(***ht*-2,4-tpcb**) (as-synthesized); Figure S7: pXRD pattern of 2(**1,2-di-I-tFb**)·(***ht*-2,4-tpcb**); Figure S8: pXRD pattern of (**1,3-di-I-tFb**)·(**2,4-bpe**); Figure S9: pXRD pattern of 2(**1,3-di-I-tFb**)·(***hh-*2,4-tpcb**); Figure S10: pXRD pattern of (**1,4-di-I-tFb**)·(**2,4-bpe**).

## Sample Availability

Samples of the compounds (**1,2-di-I-tFb**)∙(**2,4-bpe**), 2(**1,2-di-I-tFb**)∙(***ht*-2,4-tpcb**), (**1,3-di-I-tFb**)∙(**2,4-bpe**), 2(**1,3-di-I-tFb**)∙(***hh*-2,4-tpcb**), ***ht*-2,4-tpcb**, and ***hh*-2,4-tpcb** are available from the authors upon request. are available from the authors.
