# Supplementary material for: Halogen-Bond Mediated [2+2] Photodimerizations: À la Carte Access to Unsymmetrical Cyclobutanes in the Solid State
Source: Molecules. 2022 Feb 3;27(3):1048. doi: 10.3390/molecules27031048 (PMC8839528; doi:10.3390/molecules27031048)
Supplement: Supplementary file 1 [file molecules-27-01048-s001.zip › molecules-1550238-supplementary.pdf]

Supporting Information

Halogen-Bond Mediated [2+2] Photodimerizations: à la Carte Access to Unsymmetrical Cyclobutanes in the Solid State

Jay Quentin[1], Eric W. Reinheimer[2], and Leonard R. MacGillivray[1]*

[1] Department of Chemistry, University of Iowa, Iowa City, IA 52242, USA; jay-bell@uiowa.edu (J.Q.)
[2] Rigaku Americas Corporation, 9009 New Trails Drive, The Woodlands, TX 77381, USA; eric.reinheimer@rigaku.com (E.W.R.)

Department of Chemistry, University of Iowa, Iowa City, IA, 52242-1294, USA.
*Correspondence: len-macgillivray@uiowa.edu

ORCID
Jay Quentin: 0000-0002-1729-7774
Eric W. Reinheimer: 0000-0002-9491-1571
Leonard R. MacGillivray: 0000-0003-0875-677X

Supporting Information:

S1. Nuclear Magnetic Resonance (NMR) Spectroscopy Data
Figure S1. Stacked ^{1}H NMR spectra of (**1,2-di-I-tFb**)·(**2,4-bpe**) and 2(**1,2-di-I-tFb**)·(***ht*-2,4-tpcb**).
Figure S2. ^{1}H NMR spectrum of 2(**1,2-di-I-tFb**)·(***ht*-2,4-tpcb**).
Figure S3. Stacked ^{1}H NMR spectra of (**1,3-di-I-tFb**)·(**2,4-bpe**) and 2(**1,3-di-I-tFb**)·(***hh*-2,4-tpcb**).
Figure S4. ^{1}H NMR spectrum of 2(**1,3-di-I-tFb**)·(***hh*-2,4-tpcb**).

S2. Powder X-Ray Diffraction (pXRD) Data
Figure S5. pXRD pattern of (**1,2-di-I-tFb**)·(**2,4-bpe**).
Figure S6. pXRD pattern of 2(**1,2-di-I-tFb**)·(***ht*-2,4-tpcb**) (as-synthesized).
Figure S7. pXRD pattern of 2(**1,2-di-I-tFb**)·(***ht*-2,4-tpcb**).
Figure S8. pXRD pattern of (**1,3-di-I-tFb**)·(**2,4-bpe**).
Figure S9. pXRD pattern of 2(**1,3-di-I-tFb**)·(***hh*-2,4-tpcb**).
Figure S10. pXRD pattern of (**1,4-di-I-tFb**)·(**2,4-bpe**).

S1. Nuclear Magnetic Resonance (NMR) Spectroscopy Data

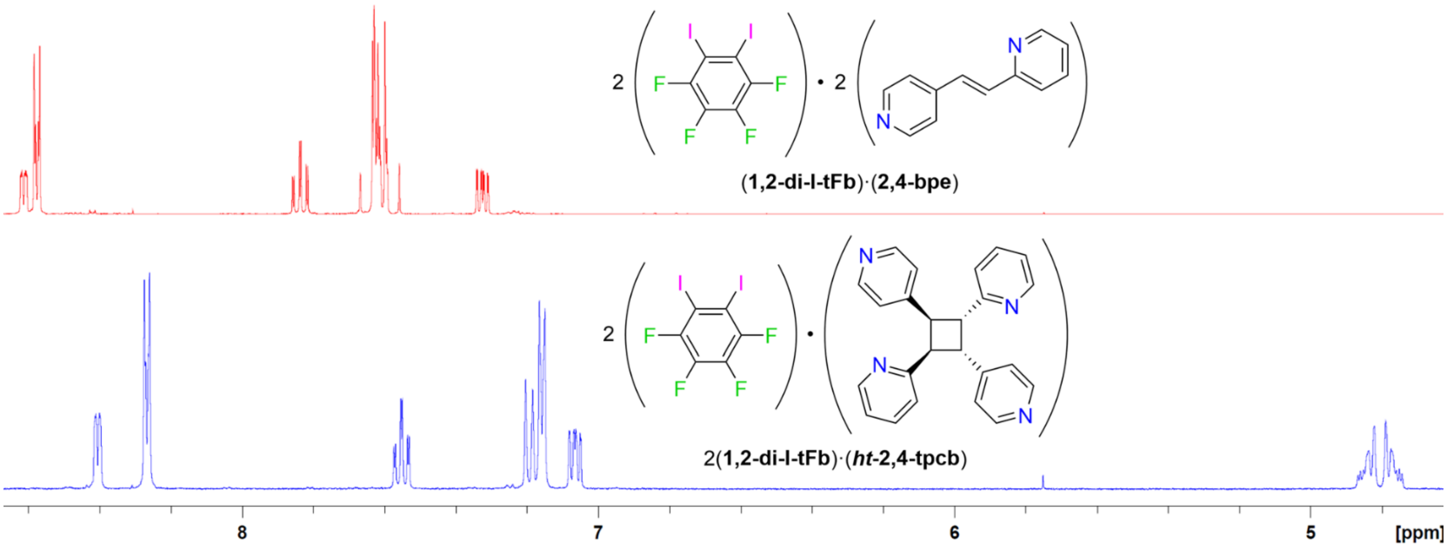

Figure S1. ^{1}H NMR (400 MHz, DMSO-d_6) spectra of (**1,2-di-I-tFb**)·(**2,4-bpe**) (top) and 2(**1,2-di-I-tFb**)·(***ht*-2,4-tpcb**) (bottom). Note complete disappearance of the pair of alkene doublets (δ_H = 7.65, 7.59 ppm) and emergence of a pair of cyclobutane resonances (δ_H = 4.87-4.82, 4.79-4.74 ppm) following UV-irradiation (100 h).

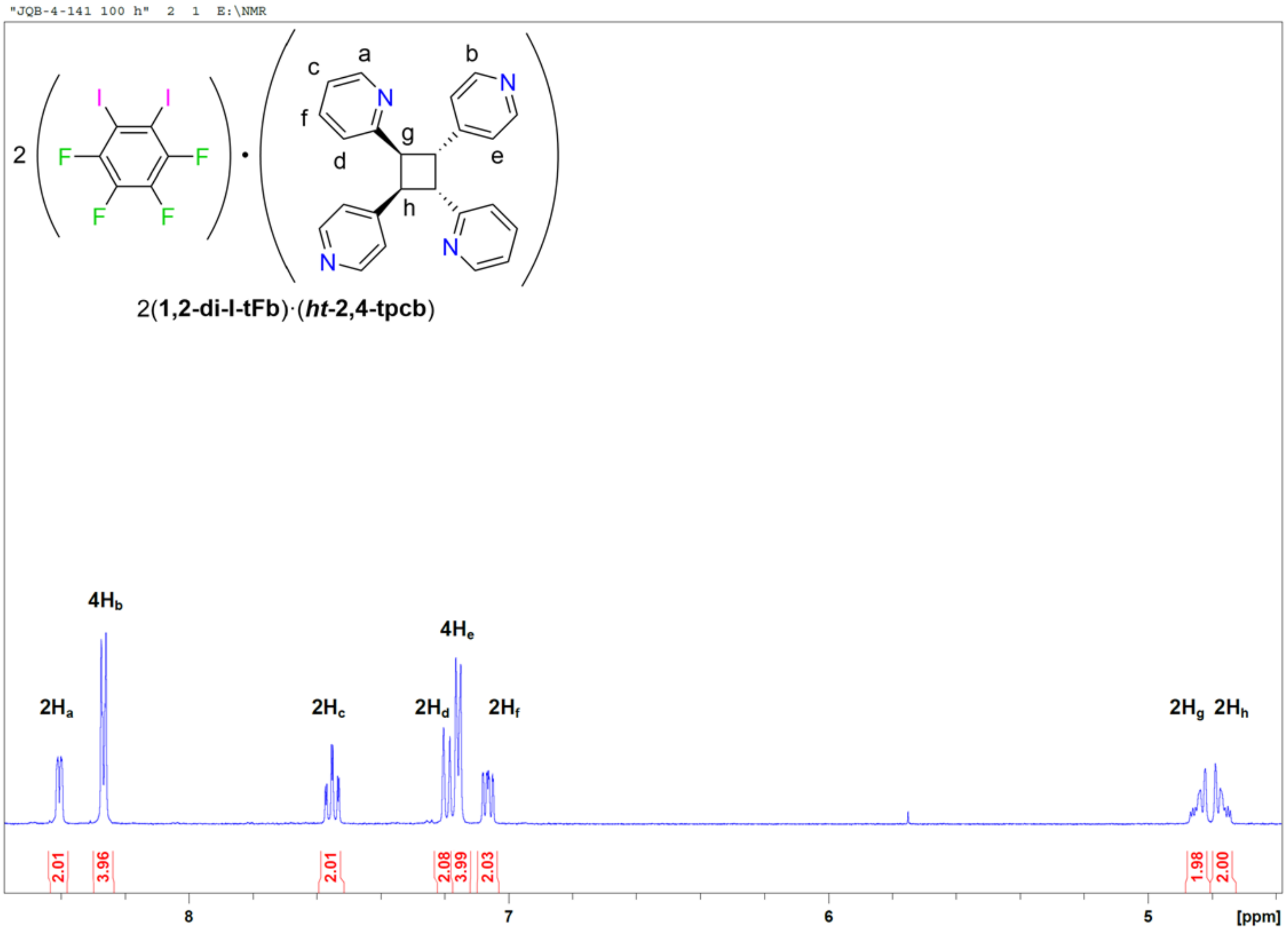

Figure S2. ^{1}H NMR (400 MHz, DMSO-d_6) spectrum of 2(**1,2-di-I-tFb**)·(***ht*-2,4-tpcb**). The horizontal axis is calibrated relative to DMSO.

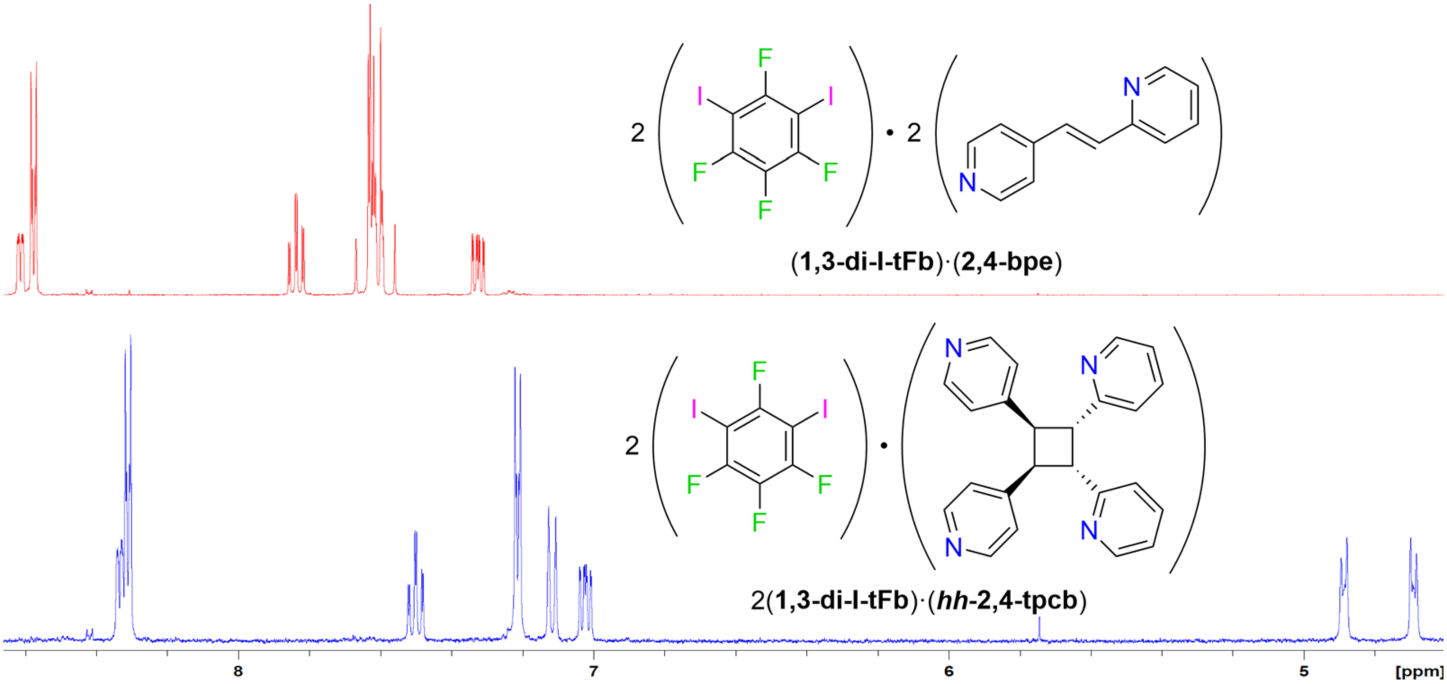

Figure S3. ^{1}H NMR (400 MHz, DMSO-d_6) spectra of (**1,3-di-I-tFb**)·(**2,4-bpe**) (top) and 2(**1,3-di-I-tFb**)·(***hh*-2,4-tpcb**) (bottom). Note complete disappearance of the pair of alkene doublets (δ_H = 7.65, 7.59 ppm) of **2,4-bpe** and the emergence of a pair of cyclobutane resonances (δ_H = 4.89, 4.69 ppm) following UV-irradiation (80 h).

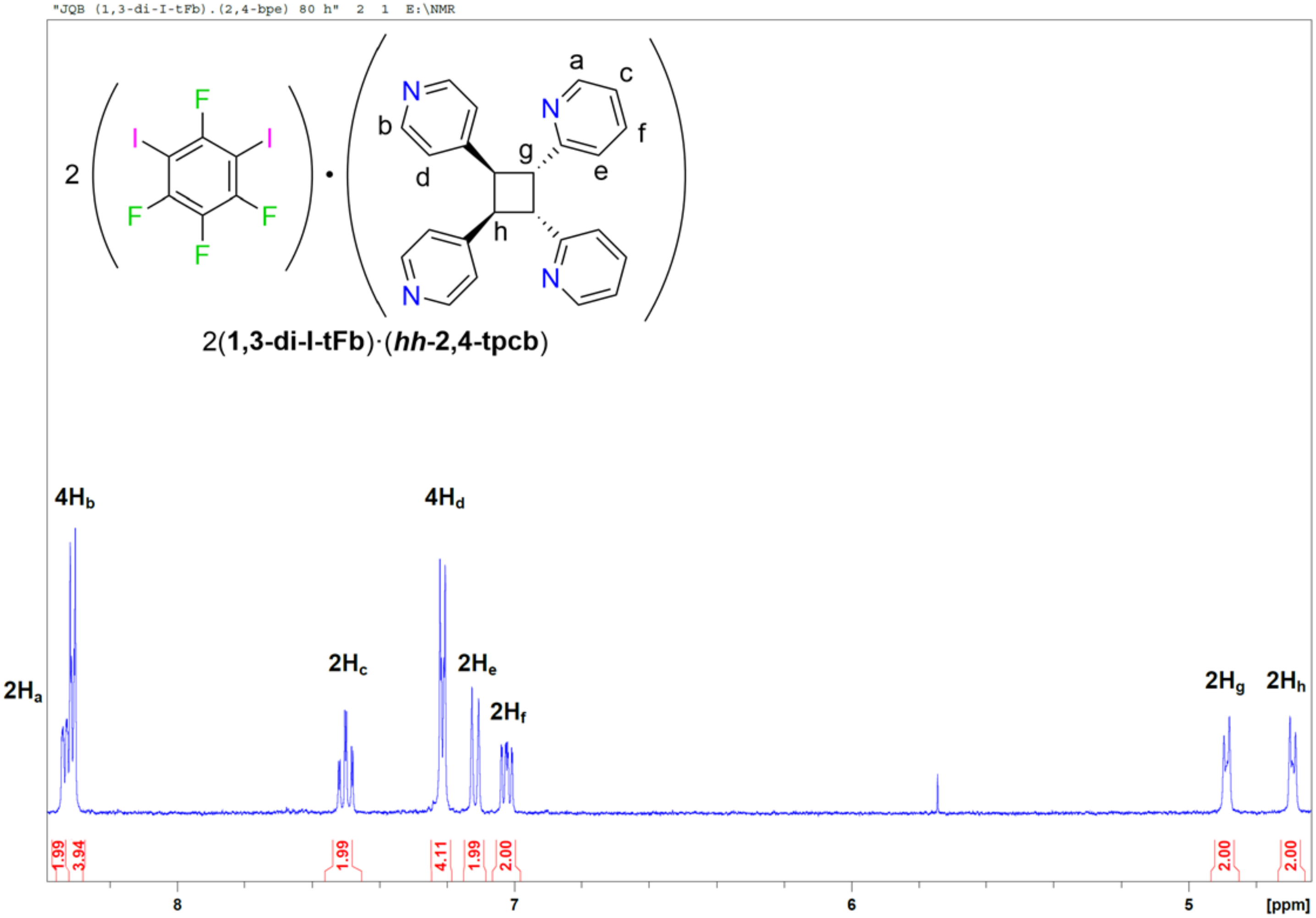

Figure S4. ^{1}H NMR (400 MHz, DMSO-d_6) spectrum of 2(**1,3-di-I-tFb**)·(***hh*-2,4-tpcb**). The horizontal axis is calibrated relative to DMSO.

S2. Powder X-Ray Diffraction (pXRD) Data

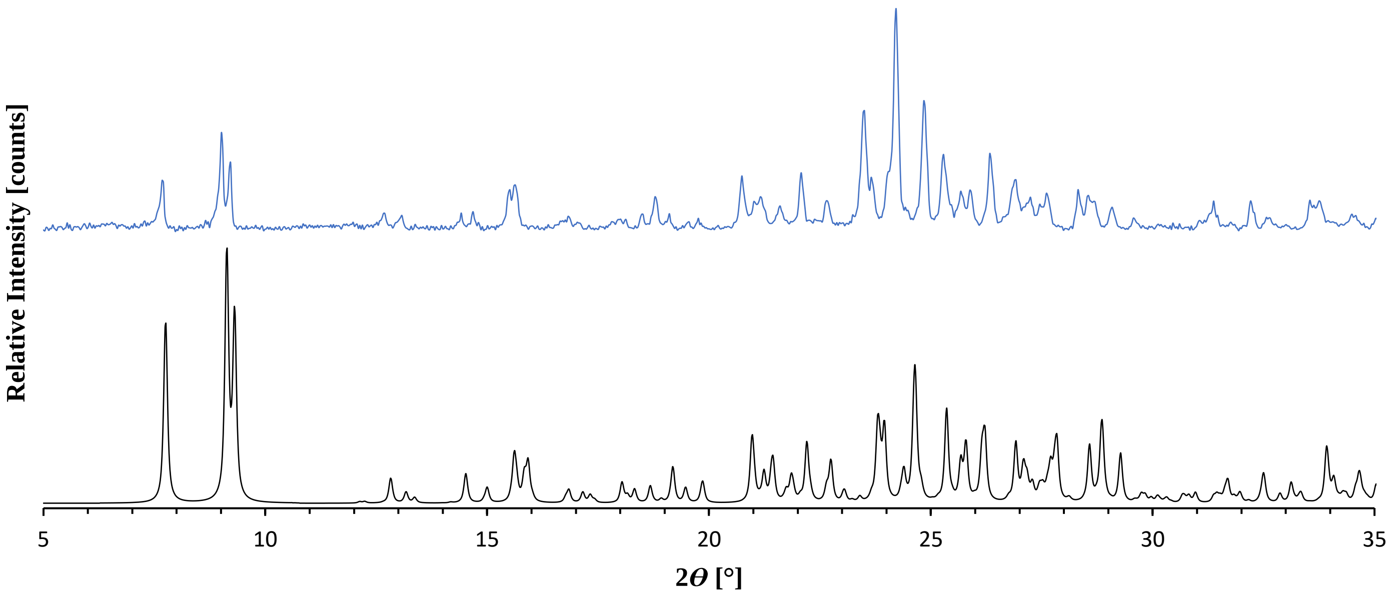

Figure S5. Experimental (top) and simulated (bottom) pXRD traces for (**1,2-di-I-tFb**)·(**2,4-bpe**).

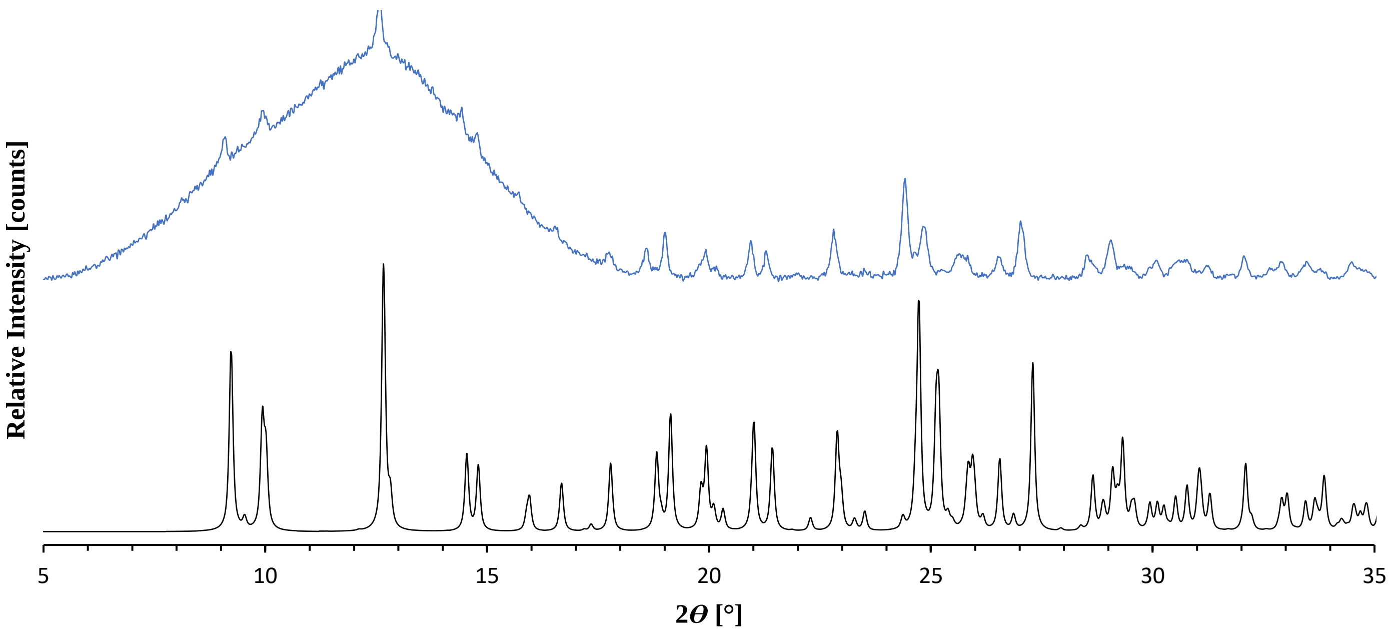

Figure S6. Experimental (top) and simulated (bottom) pXRD traces for 2(**1,2-di-I-tFb**)·(***ht*-2,4-tpcb**). Grinding to provide powder resulted in partial formation of paste and loss of crystallinity.

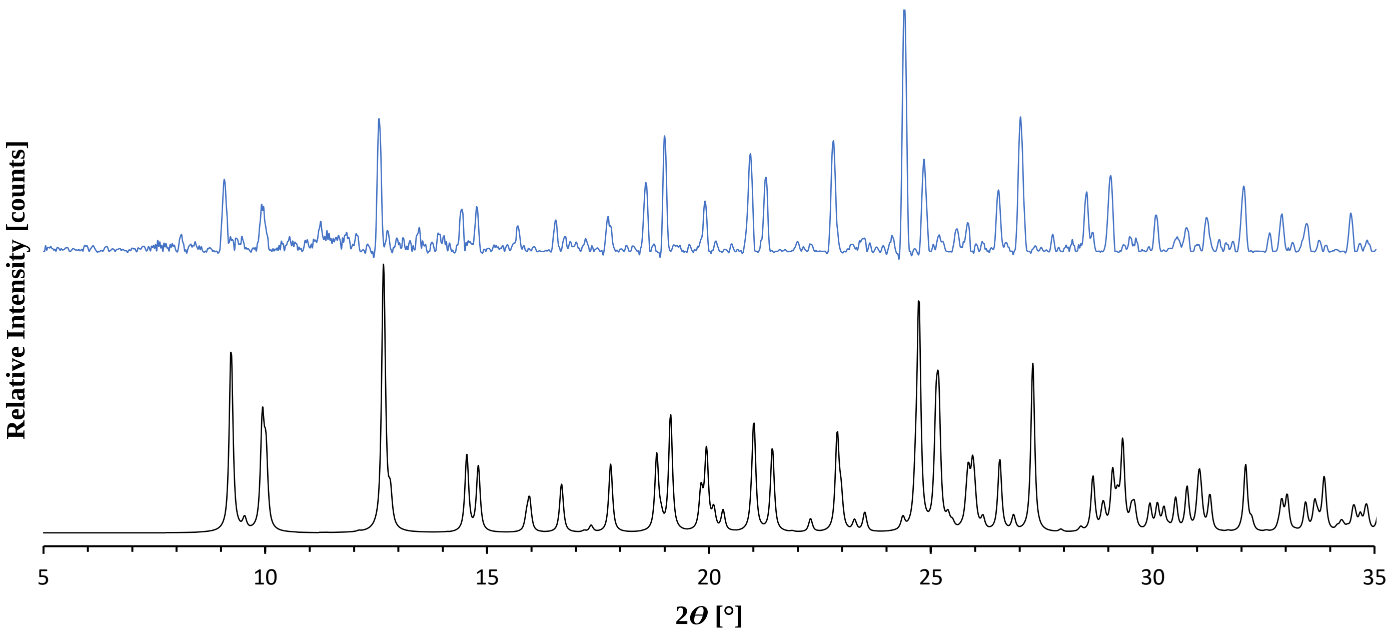

Figure S7. Experimental (top) (background subtracted) and simulated (bottom) pXRD traces for 2(**1,2-di-I-tFb**)·(***ht*-2,4-tpcb**).

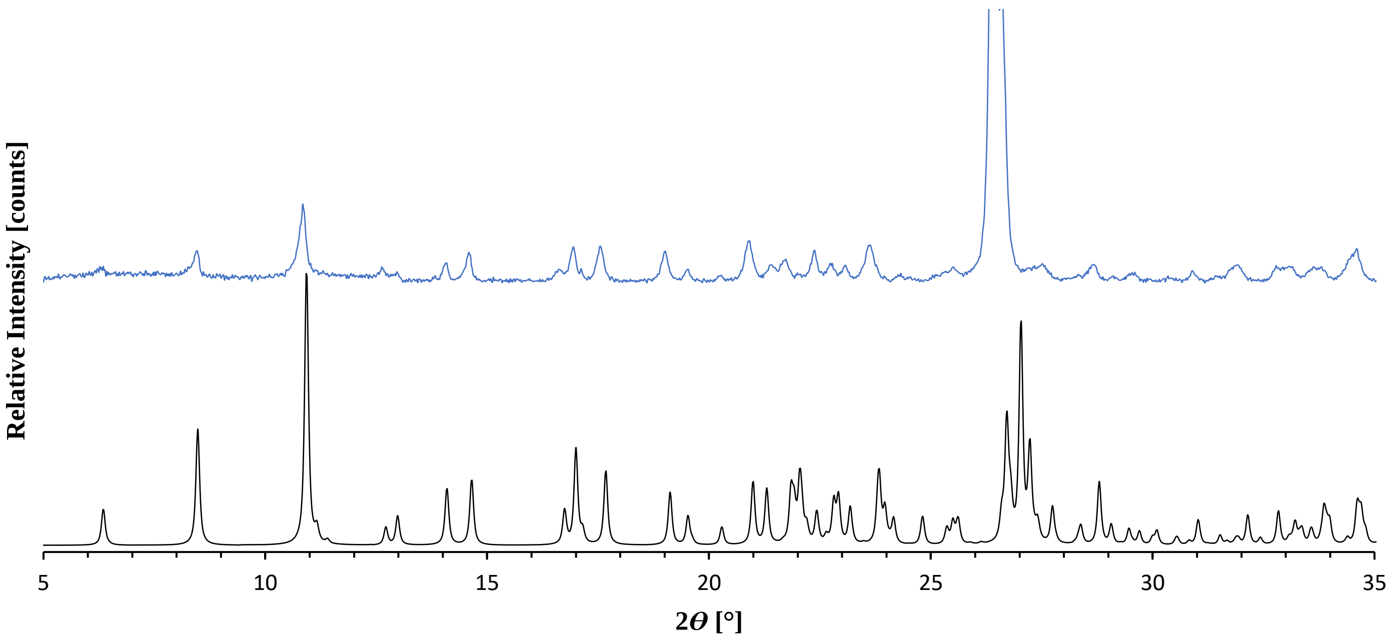

Figure S8. Experimental (top) and simulated (bottom) pXRD traces for (**1,3-di-I-tFb**)·(**2,4-bpe**).

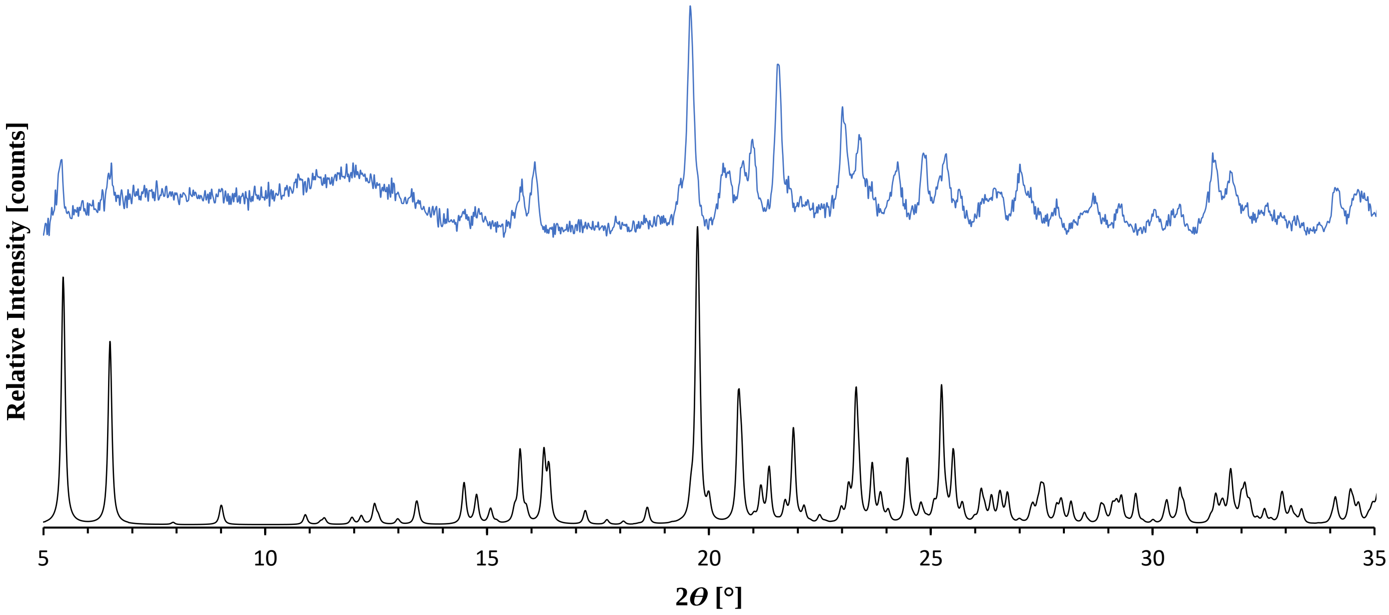

Figure S9. Experimental (top) and simulated (bottom) pXRD traces for 2(**1,3-di-I-tFb**)·(***hh*-2,4-tpcb**).

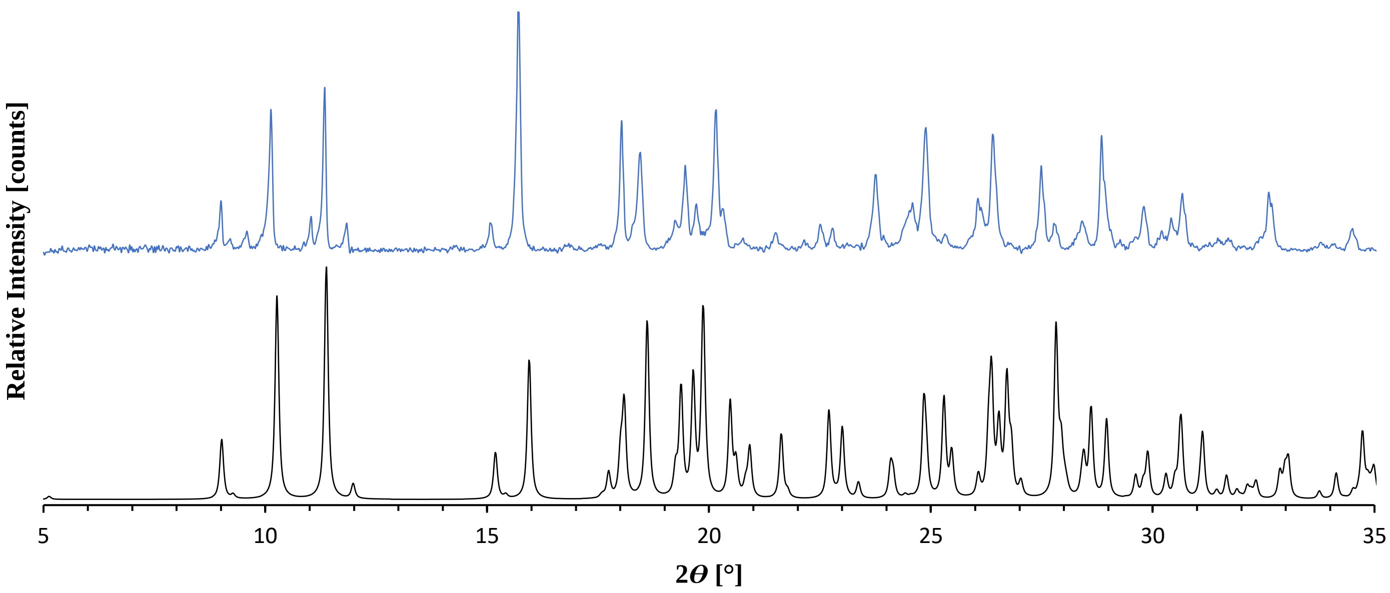

Figure S10. Experimental (top) and simulated (bottom) pXRD traces for (**1,4-di-I-tFb**)·(**2,4-bpe**).
